# Hypoxic TCs-preconditioned MSCs ameliorate acute lung injury via enhanced Treg recruitment and function through CXCL5/6-CXCR1 axis

**DOI:** 10.1186/s13287-025-04858-6

**Published:** 2025-12-26

**Authors:** Luoyue Yin, Xu Zhang, Yile Zhou, Huihui Ju, Youwei Zhu, Rongrong Gao, Pinwen Wu, Hao Fang

**Affiliations:** 1 Department of Anaesthesiology, Zhongshan Hospital, Fudan University, 180 Fenglin Road, Shanghai, 200032 China; 2 Department of Anaesthesiology, Shanghai Geriatric Medical Center, Shanghai, 201104 China; 3 NHC Key Lab of Reproduction Regulation, Shanghai Engineering Research Center of Reproductive Health Drug and Devices, Shanghai Institute for Biomedical and Pharmaceutical Technologies, Shanghai, 200237 China; 4 Shanghai-MOST Key Laboratory of Health and Disease Genomics, NHC Key Lab of Reproduction Regulation, Shanghai Institute for Biomedical and Pharmaceutical Technologies, Shanghai, 200237 China; 5 Clinical Center of Bio-Therapy at Zhongshan Hospital & Institutes of Biomedical Sciences, Shanghai Public Health Clinical Center, Fudan University, Shanghai, China; 6 Clinical Center for Biotherapy at Zhongshan Hospital, Fudan University, Shanghai, 200032 China; 7 Department of Anesthesiology, Minhang Hospital, Fudan University, Shanghai, China

**Keywords:** Acute lung injury, Mesenchymal stem cells, Telocytes, Regulatory T cells

## Abstract

**Background:**

Acute lung injury/acute respiratory distress syndrome (ALI/ARDS) remains a critical respiratory condition with limited effective treatments.

**Methods:**

This study investigated whether mesenchymal stem cells (MSCs) preconditioned with supernatant from hypoxia-cultured telocytes (TCs) could enhance therapeutic efficacy in ALI through regulatory T cell (Treg) modulation.

**Results:**

MSCs preconditioned with 5% hypoxic TC supernatant demonstrated superior efficacy in ameliorating LPS-induced lung injury compared to conventional MSCs or TC monotherapy, as evidenced by preserved alveolar architecture, reduced inflammatory infiltration, and decreased pro-inflammatory cytokines. Mechanistically, these preconditioned MSCs significantly enhanced Treg recruitment to injured lung tissues and improved their immunosuppressive function through the CXCL5/6-CXCR1 axis, an effect that was substantially attenuated upon siRNA-mediated disruption of this pathway, and was further corroborated in a humanized ALI mouse model where preconditioned-MSC treatment improved survival, reduced lung injury severity, and enhanced Treg recruitment and function in a CXCL5/6 signaling-dependent manner.

**Conclusions:**

These findings reveal a novel mechanism by which hypoxic TC supernatant enhances MSC therapeutic efficacy in ALI through the CXCL5/6-CXCR1 axis, providing a promising strategy for optimizing cellular therapy in inflammatory pulmonary disorders.

**Supplementary Information:**

The online version contains supplementary material available at 10.1186/s13287-025-04858-6.

## Introduction

Acute lung injury/acute respiratory distress syndrome (ALI/ARDS) is a severe critical respiratory condition characterized by capillary leakage, impaired gas exchange, and hypoxemia due to uncontrolled pulmonary inflammation [1, 2]. Although modern medical treatments primarily focus on anti-infection therapy and mechanical ventilation, their effectiveness in reducing ARDS mortality remains limited [3]. Mesenchymal stem cells (MSCs), owing to their low immunogenicity and multipotent differentiation capabilities, have emerged as a promising therapeutic avenue for ALI/ARDS [4, 5]. Their therapeutic potential is particularly associated with immunomodulatory properties, especially the ability to regulate Treg/Th17 homeostasis - a key mechanism underlying MSC-mediated immune regulation [6]. However, the clinical efficacy of MSCs remains inconsistent, with approximately 60% of clinical trials being terminated or deemed ineffective. This variability may be attributed to factors such as passage-dependent changes during in vitro expansion and the influence of the inflammatory microenvironment in vivo. Thus, there is an urgent need to optimize MSC-based treatment strategies.

Telocytes (TCs) are a novel type of interstitial cell widely distributed in the alveolar interstitium, small blood vessels, and airway smooth muscle layers [7]. They secrete multiple repair-related factors and play an important role in tissue repair and inflammatory regulation. A synergistic interaction exists between TCs and MSCs [8–10], wherein TCs not only enhance MSC proliferation and activity but also augment their immunomodulatory functions, thereby potentiating MSC-mediated therapeutic effects in inflammatory environments [10]. Notably, TCs significantly promote the migration and tissue-specific homing of MSCs to injured lung tissue, which is critical for mitigating pulmonary edema and enhancing alveolar fluid clearance.

As key modulators of MSC-mediated immunoregulation, regulatory T cells (Tregs) function to suppress excessive inflammation and maintain immune equilibrium. Studies have demonstrated that Tregs inhibit the activation and proliferation of effector T cells by secreting immunosuppressive cytokines (e.g., IL-10, TGF-β) and expressing specific surface molecules (e.g., CTLA-4, PD-1), thereby limiting inflammatory injury [11]. In ALI, the number of Tregs in injured lung tissues is significantly reduced, accompanied by a marked impairment in their immunoregulatory function, leading to exacerbated inflammation and aggravated lung injury [12, 13]. Despite the therapeutic potential of enhancing Treg recruitment and function at inflammatory sites, a key challenge remains in achieving targeted migration and sustained anti-inflammatory efficacy while maintaining their stability in complex inflammatory microenvironments.

This study aims to elucidate the mechanistic basis through which TCs enhance the capacity of MSCs to promote Treg recruitment and function in ALI, with particular focus on their immunomodulatory effects in the inflammatory pulmonary microenvironment.

## Materials and methods

The work has been reported in line with the ARRIVE guidelines 2.0.

### Isolation and culture of Tregs

Human peripheral blood mononuclear cells (PBMCs) were isolated from peripheral blood by density gradient centrifugation and quantified using an automated cell counter. CD4^+^CD25^+^ Tregs were isolated using magnetic-activated cell sorting (MACS; Stemcell Technologies, #18023) according to the manufacturer’s instructions. To enhance Treg purity, the isolated cells were cultured in serum-free X-VIVO-15 medium (Lonza, #04-418Q) in plates containing pre-seeded MSCs that were either conventionally cultured, T-cell stimulated, or siRNA-treated. Tregs were simultaneously activated and expanded by supplementing the culture with Dynabeads™ Human T-Activator CD3/CD28 (Thermo Fisher Scientific, #11456D) at a 1:1 bead-to-cell ratio and recombinant human IL-2 (1000 IU/mL; ACROBiosystems, #GMP-L02H14).

### Isolation and culture of TCs

Primary human pulmonary TCs were isolated from discarded lung tissue specimens obtained during surgical procedures using a sequential adhesion-based isolation method. Briefly, minced lung tissue was enzymatically digested, and the resulting cell suspension was subjected to differential adhesion steps to enrich for TCs. Flow cytometry-based cell sorting was performed to obtain a purified population of CD34^+^PDGFRα^+^vimentin^+^ TCs. The sorted cells were maintained in culture medium supplemented with anti-CD34 (Clone EP373Y, Abcam, #ab81289), anti-PDGFRα (Clone 16A1, BioLegend, #323506), and anti-vimentin (Clone V9, Santa Cruz Biotechnology, #sc-6260) antibodies at manufacturer-recommended concentrations.

### TC-supernatant stimulation of MSCs

TCs were cultured in MSC-specific complete medium for 72 h. The culture supernatant was harvested and centrifuged at 800 g for 5 min to remove cellular debris, yielding TC- supernatant. MSCs were then cultured for 72 h in a 1:1 mixture of this supernatant and fresh MSC-specific complete medium. TC-MSC and 5% TC-MSC referred to MSCs pre-conditioned with supernatant from TCs cultured under normoxic (21% O₂) or hypoxic (5% O₂) conditions, respectively.

### Real-time cytotoxicity assays

CAR-T-CD19 cells were prepared as previously described [27]. NCI-H292-CD19 cells (1 × 10^4^) were seeded in 150 µL of R10 medium in xCELLigence 96-well flat-bottom plates (ACEA Biosciences, San Diego, USA) and incubated overnight to establish baseline cell proliferation parameters. CAR-T-CD19 cells were subsequently added at a 1:1 effector-to-target ratio, and Treg cells were added at a 2:1 ratio relative to CAR-T-CD19 cells. T cell-mediated cytotoxicity was monitored as changes in electrical impedance measured at 15-minute intervals for 45 h using the xCELLigence^®^ RTCA SP device according to the manufacturer’s instructions.

### Proliferation Inhibition assay

CD8^+^ T cells were isolated from fresh or cryopreserved PBMCs and immediately labeled with CellTrace eFluor670 proliferation dye (Invitrogen, #00-5523) according to the manufacturer’s instructions. Labeled cells were washed twice with PBS and plated in 96-well U-bottom plates (1 × 10^5^ cells per well) in T cell medium supplemented with IL-2. CD3/CD28 beads were added at a 1:10 bead-to-cell ratio to stimulate proliferation. Tregs or control Tregs were added to the culture, and the co-culture was maintained for 96 h.

All experimental conditions were performed in triplicate, and replicate wells were combined prior to flow cytometric analysis. To minimize competition for cytokines that could confound proliferation assessment, suppressor cells were rested in culture for 72–96 h after CD3/CD28 bead stimulation and then incubated in cytokine-free T cell medium for 24 h before co-culture with responder cells.

### LPS-induced lung injury model

Lung injury was induced by intranasal administration of lipopolysaccharide (LPS) at 5 mg/kg for C57BL/6 mice or 2.5 mg/kg for NOG mice (Sigma-Aldrich, Cat# L2880). At predetermined time points following LPS treatment, mice were euthanized and tissues were collected as follows: mice were euthanized by overdose anesthesia induced via intraperitoneal injection of sodium pentobarbital (150 mg/kg; Sigma-Aldrich, Cat# P3761) prepared in sterile saline (10 mg/mL). Adequate anesthetic depth was confirmed by the absence of a toe-pinch reflex. The thoracic cavity was then opened and a perfusion needle was inserted into the left ventricle via cardiac puncture. Transcardial perfusion was performed using phosphate-buffered saline (PBS, pH 7.4; Gibco™, Cat# 10010023). Lung tissues were subsequently harvested for histopathological evaluation by hematoxylin and eosin (H&E) staining. Lung injury assessment was shown in Supplementary Materials.

### Statistical analysis

Statistical analyses were performed using GraphPad Prism version 8 (GraphPad Software). All data are presented as mean ± standard deviation (SD). Statistical comparisons between two groups were made using unpaired t-tests. One-way ANOVA with appropriate post-hoc tests was used for comparisons involving three or more groups. Data were transformed when necessary to normalize variance. *p < 0.05; **p < 0.01; ***p < 0.001, ****p < 0.0001.

## Results

### Combined TC and MSC therapy significantly ameliorated ALI lung injury and enhanced the immunosuppressive function of Tregs

**Fig. 1:**
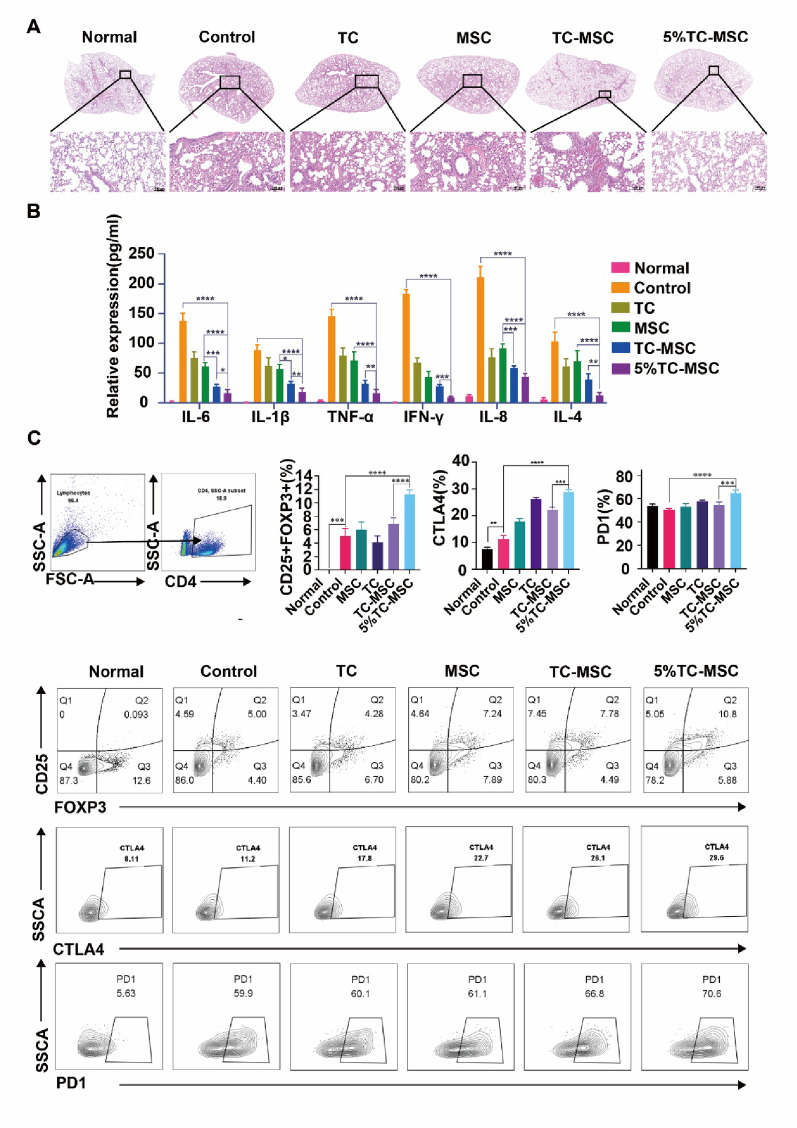
Combined therapy with TCs and MSCs ameliorated lung injury severity in ALI mouse model and enhanced the immunosuppressive function of Tregs. **A** Histological analysis of lung tissues from different groups. H&E staining showed the pathological changes in lung tissues from normal mice (Normal), PBS-treated ALI mice (Control), TC-treated ALI mice (TC), MSC-treated ALI mice (MSC), ALI mice treated with MSCs pre-conditioned with supernatant collected from TCs (TC-MSC), and ALI mice treated with MSCs pre-conditioned with supernatant collected from 5% hypoxia-cultured TCs (5% TC-MSC). Scale bar = 100 μm. **B** ELISA detection of inflammatory cytokine expression levels in bronchoalveolar lavage fluid (BALF) from different groups, including IL-6, IL-1β, TNF-α, IFN-γ, IL-8, and IL-4. *N* = 6. **p < 0.01, ***p < 0.001, ****p < 0.0001. **C** Flow cytometric analysis of Tregs proportion and functional marker expression in lung tissues from different groups. Upper: statistical analysis of CD4^+^CD25^+^FOXP3^+^ Tregs percentage, CTLA4^+^ cell percentage, and PD1^+^ cell percentage. Below: representative flow cytometric plots showing CD25^+^FOXP3^+^ cells, CTLA4 and PD1 expression in each group. *N* = 6. **p < 0.01, ***p < 0.001, ****p < 0.0001

The protective effects of combined TC and MSC therapy on ALI-induced lung injury were first evaluated in this study. To clarify the experimental system, it should be noted that in this study, “TC-MSC” specifically refers to MSCs cultured with supernatant collected from TC cultures, which were then exclusively co-cultured with Tregs without direct TC involvement. MSCs preconditioned with 5% hypoxic TC supernatant exhibited significantly enhanced proliferation compared to control conditions (Fig. S1). In comparison with the control group that exhibited typical ALI pathological features, both the TC-alone and MSC-alone treatment groups demonstrated moderate improvement in lung histology, though notable damage remained. In contrast, the TC-MSC combined treatment group, especially the 5% hypoxia-cultured TC-MSC group (5% TC-MSC), exhibited significant histological improvements characterized by attenuated inflammatory infiltration and preserved alveolar structure (Fig. 1A). ELISA results revealed that, compared to the control group, the levels of proinflammatory cytokines (IL-6, IL-1β, TNF-α, IFN-γ and IL-8) in the TC-MSC and 5% TC-MSC combined treatment groups were significantly lower than those in the TC-alone and MSC-alone groups (*p* < 0.01, *p* < 0.001, *p* < 0.0001) (Fig. 1B). To explore the immunoregulatory mechanisms, Treg proportions and functional marker expression in lung tissues were analyzed using flow cytometry. The results demonstrated that the percentage of CD4^+^CD25^+^FOXP3^+^ Tregs in the 5% hypoxia- cultured TC-MSC group exhibited the most pronounced increase (*p* < 0.0001), surpassing those in the TC-alone, MSC-alone and TC-MSC groups (Fig. 1C). Moreover, elevated expression of Treg functional markers CTLA4 and PD1 was also observed in the 5% hypoxia- cultured TC-MSC group (Fig. 1C).

### MSCs pre-conditioned with TCs-derived supernatant significantly enhanced Tregs phenotype and functional properties

**Fig. 2:**
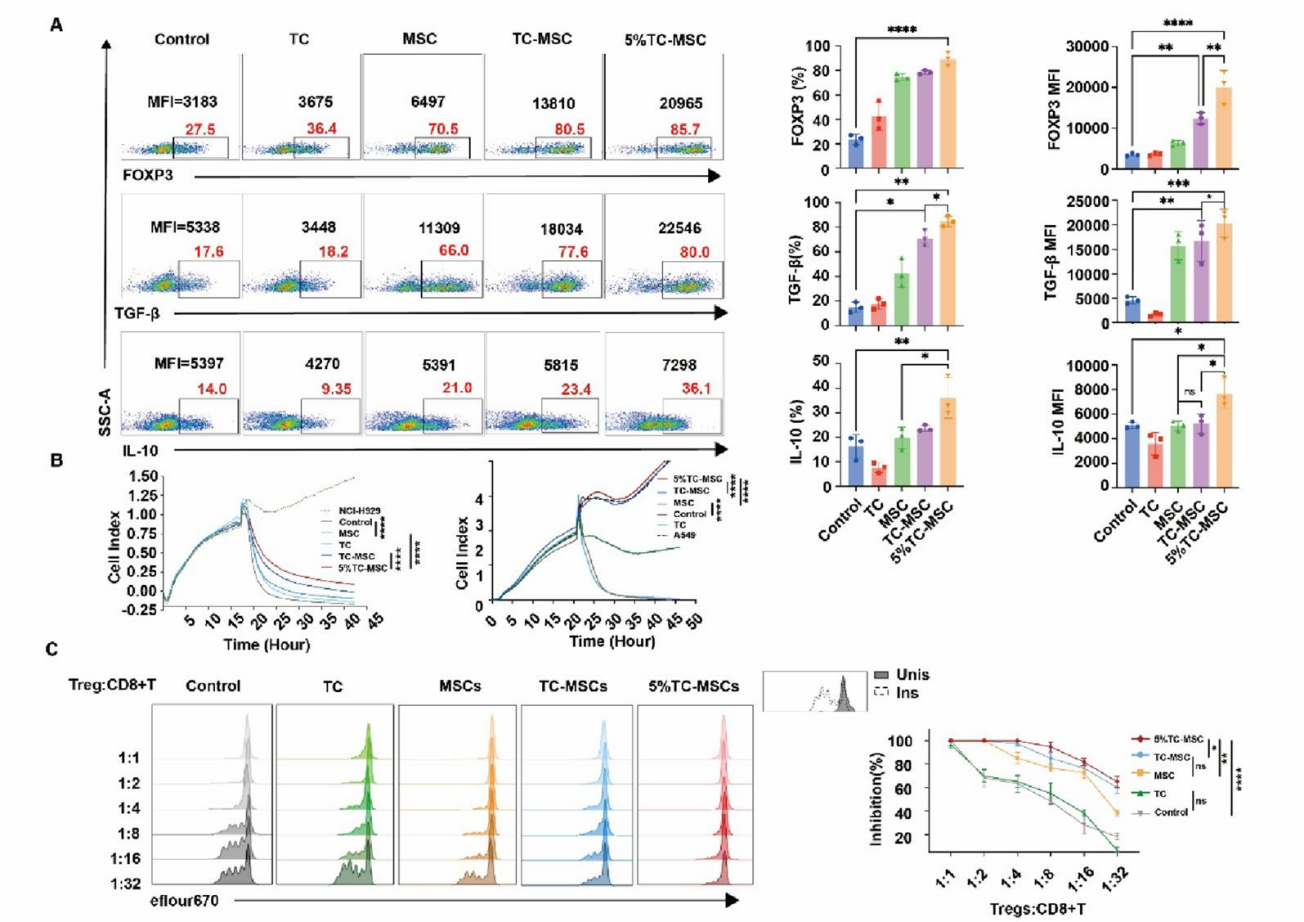
MSCs pre-conditioned with TCs-derived supernatant enhanced phenotypic and functional properties of Tregs.** A** Flow cytometric analysis of Foxp3, TGF-β, and IL-10 expression in Tregs across different treatment groups. *N* = 3.** B** RTCA measurements of Treg-mediated inhibition of CAR-T cell cytotoxicity against NCIH929 cells (left) and A549 cells (right) across different treatment groups.** C** Suppressive capacity of Tregs from different treatment groups against CD8^+^ T cell cytotoxicity. Flow cytometry plots on the left demonstrated cytotoxicity inhibition at varying Treg: CD8^+^ T cell ratios (1:1 to 1:32); the right panel showed corresponding inhibition percentage statistics. Unis denoted isolated CD8^+^ T cells without Treg co-culture or bead stimulation, while Ins referred to isolated CD8^+^ T cells with anti-CD3/CD28 bead stimulation but without Tregs. *N* = 5. *p < 0.05, **p < 0.01, ***p < 0.001, ****p < 0.0001. Treg, Tregs alone; TC, Tregs co-cultured with TCs; MSC, Tregs co-cultured with MSCs; TC-MSC, Tregs co-cultured with MSCs pre-conditioned with normoxic TC-derived supernatant; 5% TC-MSC, Tregs co-cultured with MSCs pre-conditioned with supernatant from TCs cultured under 5% hypoxic conditions

As shown in Fig. 2A, flow cytometry analysis indicated that compared with the Tregs alone group, co-culture of Tregs with TCs (TC group) showed limited enhancement of Foxp3 expression; whereas Tregs co-cultured with MSCs (MSC group) significantly enhanced Foxp3 expression (*p* < 0.05). Notably, MSCs pretreated with TC supernatant (TC-MSC group) and then co-cultured with Tregs significantly increased Foxp3 expression levels (*p* < 0.001), while MSCs pretreated with TC supernatant under 5% hypoxic conditions (5% TC-MSC group) demonstrated the most pronounced enhancement (*p* < 0.0001). Similar trends were reflected in the expression of anti-inflammatory factors TGF-β and IL-10, the 5% TC-MSC group exhibited the highest levels, significantly superior to all other groups (*p* < 0.0001).

To determine the functional correlation of these phenotypic alterations, we subsequently assessed the immunosuppressive properties of Tregs following different treatment conditions. Real-time cell analysis demonstrated that Tregs treated by the 5% TC-MSC group exhibited the strongest inhibitory effect on CAR-T cell-mediated killing of NCIH929 and A549 cells (*p* < 0.0001) (Fig. 2B). Additionally, in experiments testing Treg inhibition of CD8^+^ T cell cytotoxicity (Fig. 2C), as the ratio of Tregs to CD8^+^ T cells decreased from 1:1 to 1:32, the inhibitory effect of each group showed a decreasing trend. However, the 5% TC-MSC group maintained significant inhibition rates even at the lowest ratio, markedly higher than the TC and MSC groups (*p* < 0.01). These results suggested that the supernatant from hypoxia-cultured TCs significantly enhances MSC-mediated regulation of Tregs, as evidenced by the upregulation of key anti-inflammatory molecules and improved immunosuppressive properties of Tregs.

### Hypoxic TC supernatant-conditioned MSCs enhanced Treg recruitment and function through CXCL5/6-CXCR1 axis

**Fig. 3:**
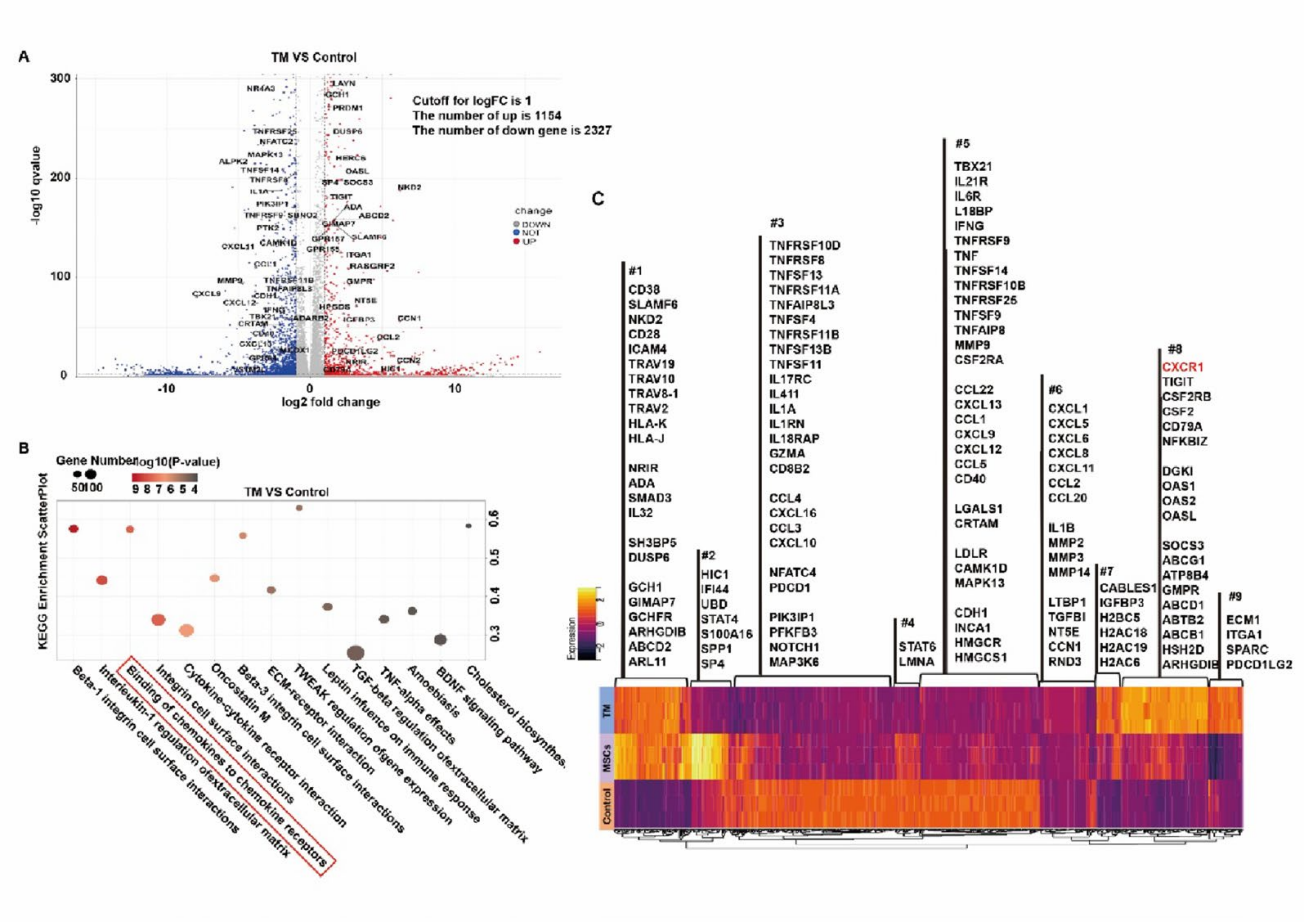
Transcriptomic profiling of iTregs regulated by MSCs pre-conditioned with supernatant from TCs cultured under 5% hypoxic conditions.** A** Volcano plot of differentially expressed genes (DEGs) in iTregs co-cultured with MSCs pre-conditioned by supernatant from TCs cultured under 5% hypoxic conditions (TM group), compared to the blank medium (Control group).** B** KEGG pathway enrichment analysis of DEGs between TM-treated and control iTregs.** C** Hierarchical clustering heatmap of the top DEGs showed distinct gene expression patterns between TM-treated and control iTregs across 9 gene clusters (#1-#9)

To investigate the molecular mechanisms underlying enhanced Treg function mediated by MSCs pre-treated with hypoxic TC supernatant, we performed transcriptomic analysis of iTregs co-cultured with MSCs pre-conditioned with supernatant from TCs cultured under 5% hypoxic conditions (TM group) versus blank medium controls. RNA-seq revealed 1,154 upregulated and 2,237 downregulated genes in TM-treated iTregs versus controls (Fig. 3A). Significantly upregulated genes in TM-treated Tregs included *Foxp3*, *Tigit*, *Cd73*, and *Hic1*, which are markers of Treg functionality and stability, as well as *Socs3*, *Dusp*, *Ras*, and *Oasl*, associated with cell proliferation and activation. Downregulated genes included members of the *Tnf* family, inflammatory chemokines, *Mmp*s, and *Ifng*, indicating a shift away from pro-inflammatory programs. KEGG enrichment analysis identified several immunoregulatory pathways, particularly the cytokine-cytokine receptor interaction pathway, along with chemokines to chemokine receptors, TNF-α effects, TGF-β regulation of extracellular matrix, and integrin cell surface interactions, suggesting a profound transcriptional reprogramming toward immune regulation (Fig. 3B). Clustering of top DEGs showed that clusters #1, #7, #8, and #9 were enriched in genes associated with Treg function (*Foxp3*, *Cd38*, *Tigit*, *Cd79*, *Hla*, *Samd3*, *Ada*, *Sparc*), surface receptors and adhesion molecules (*Trav*, *Icam*), markers of proliferation and metabolism (*Cd28*, *Socs3*, *Gtp*, *Atp5*), and early response genes (*Oas*, *Sh3*, *Dusp*). Conversely, TM-treated iTregs exhibited downregulation of inflammatory cytokines (*Tnf*, *Mmp*s, *Il1*, *Il17*) and components of inflammatory signaling pathways (*Pik3*, *Stat*, *Mapk*, *Hmgcr*, *Nfkb*). Notably, compared to the control group, Tregs in the TM group showed significantly increased expression of the chemokine receptor *Cxcr1*, indicating enhanced chemotactic potential (Fig. 3C).

**Fig. 4:**
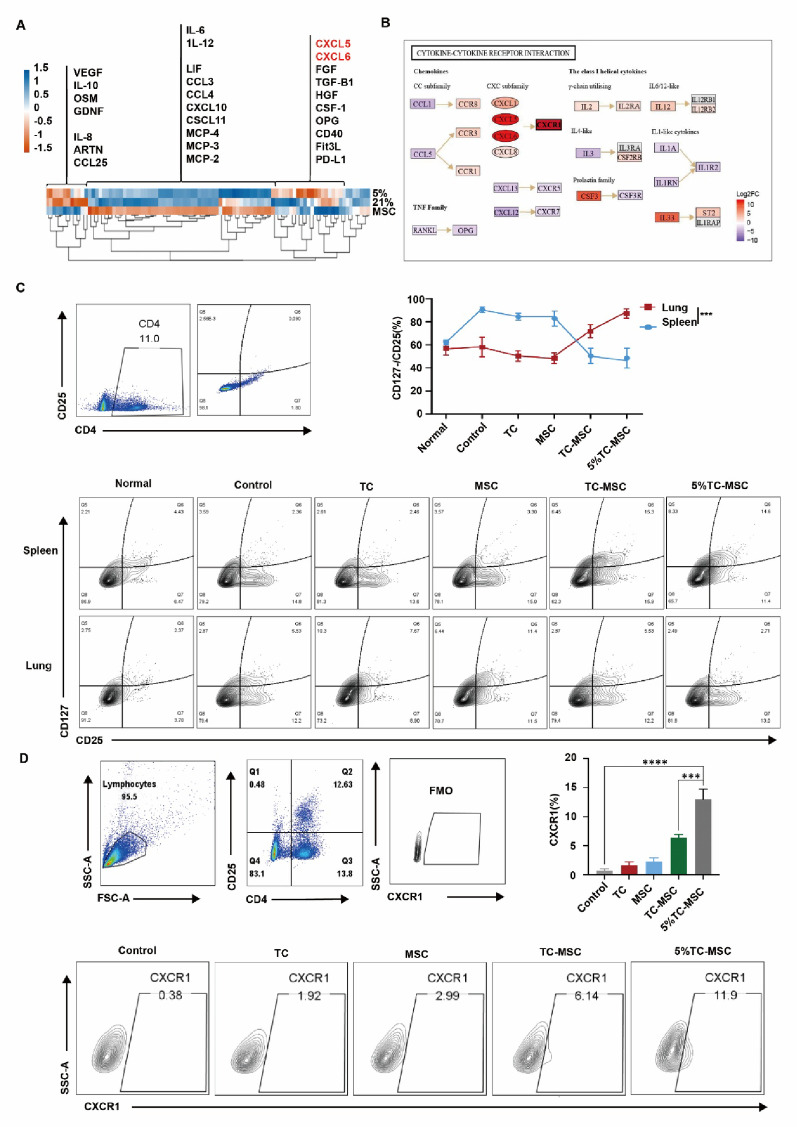
Telocytes enhanced MSC-mediated Treg recruitment through the CXCL5/6-CXCR1 axis.** A** Olink proteomic analysis comparing the supernatant of MSCs pre-conditioned with supernatant from normoxia-cultured TCs (21% O₂) versus those pre-conditioned with supernatant from hypoxia-cultured TCs (5% O₂). **B** Integrated analysis combining MSC secretome (Olink proteomics) and Treg transcriptome (RNA-seq) to predict chemokine-receptor interactions.** C** Flow cytometry analysis of CD4^+^CD25^+^CD127^−^ Treg distribution in lung and spleen tissues of normal mice (Normal), PBS-treated ALI mice (Control), TC-treated ALI mice (TC), MSC-treated ALI mice (MSC), ALI mice treated with MSCs pre-conditioned with normoxic TC supernatant (TC-MSC), ALI mice treated with MSCs pre-conditioned with hypoxic TC supernatant (5% TC-MSC). *N* = 6. ***p < 0.001. **D** Flow cytometry analysis of CXCR1 expression on Tregs in lung tissues of PBS-treated ALI mice (Control), TC-treated ALI mice (TC), MSC-treated ALI mice (MSC), ALI mice treated with MSCs pre-conditioned with normoxic TC supernatant (TC-MSC), ALI mice treated with MSCs pre-conditioned with hypoxic TC supernatant (5% TC-MSC). *N* = 6. ***p < 0.001, ****p < 0.0001

To investigate the molecular mechanisms through which TCs enhance MSC-mediated Treg recruitment, we performed Olink proteomic analysis of MSC supernatant. Compared to MSCs preconditioned with normoxic TC supernatant, those treated with hypoxic TC supernatant (5% O₂) showed significantly upregulated secretion of multiple cytokines, particularly CXCL5 and CXCL6 (Fig. 4A). The selective upregulation of CXCL5 and CXCL6 observed against a general trend of chemokine downregulation suggested their potentially significant functional involvement, meriting further investigation in this study. Bioinformatics analysis pairing DEPs in MSC supernatant with DEGs in Tregs revealed a critical ligand-receptor interaction between MSC-derived CXCL5/CXCL6 and Treg-expressed CXCR1, suggesting the functional significance of the CXCL5/6-CXCR1 axis in this process (Fig. 4B). Next, we administered different TC- and MSC-based treatments to ALI model mice and analyzed the distribution of CD4^+^CD25^+^CD127^−^ Tregs in spleen and lung tissues. Flow cytometry analysis demonstrated that pulmonary Treg proportions were comparable to controls in both TC and MSC groups, while the TC-MSC group showed significantly increased pulmonary Treg accumulation, with the highest percentage in the 5% TC-MSC group (*p* < 0.001), contrasting with reduced splenic Treg levels. Splenic Treg percentages were significantly lower in the 5% TC-MSC group (Fig. 4C), indicating that MSCs pre-conditioned with hypoxic TC supernatant promoted peripheral Treg migration to inflamed lungs. We further examined CXCR1 expression on pulmonary Tregs and found that Tregs in the 5% TC-MSC group exhibited the highest CXCR1 expression (*p* < 0.0001), followed by MSCs pre-conditioned with normoxic TC supernatant, while TC and MSC groups showed slight increases compared to controls (Fig. 4D). These findings indicated that hypoxia-cultured TCs enhance MSC secretion of CXCL5/6, thereby promoting CXCR1 expression on Tregs and ultimately strengthening Treg recruitment to inflammatory lung tissues.

**Fig. 5:**
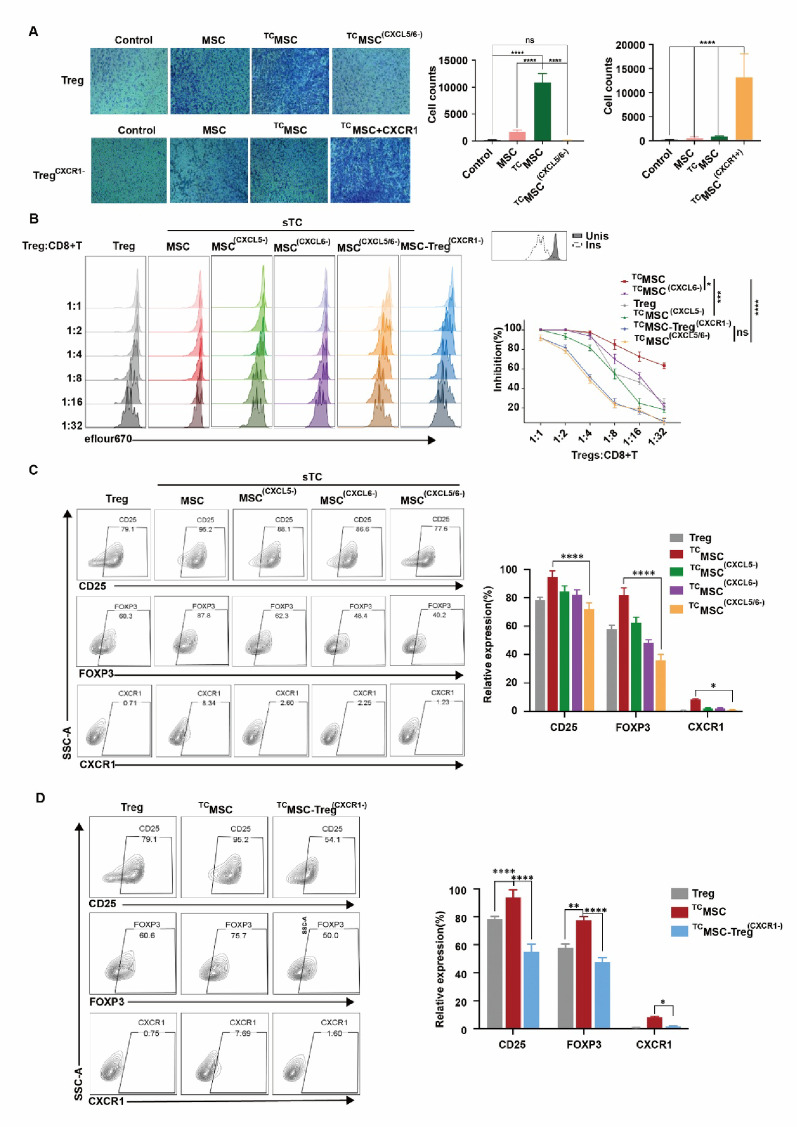
Disruption of the CXCL5/6-CXCR1 axis significantly inhibited Treg migration and immunosuppressive function induced by MSCs pre-conditioned with hypoxic TC supernatant. **A** Transwell migration assay evaluating the chemotactic capacity of Tregs under different conditions. Upper: The chemotactic effects on Tregs were compared across different conditions: Tregs cultured alone (Control), Tregs co-cultured with TCs (TC), Tregs co-cultured with MSCs (MSC), and Tregs cocultured with MSCs pretreated with hypoxic TC supernatant (^TC^MSC), including a parallel group where MSCs underwent additional siRNA-mediated CXCL5/6 knockdown (^TC^MSC^CXCL5/6−^). Lower: The chemotactic responses of CXCR1-knockdown Tregs (Tregs^CXCR1−^) were assessed under different conditions: cultured alone (Control), co-cultured with TCs (TC), co-cultured with MSCs (MSC), or co-cultured with MSCs pretreated with hypoxic TC supernatant (^TC^MSC), with an additional group where CXCR1 was re-expressed in these Tregs (TC-MSC + CXCR1). *N* = 6. **p < 0.01, ***p < 0.001, ****p < 0.0001. **B** Suppression assay comparing the immunosuppressive capacity of Tregs cultured alone (Treg), Tregs co-cultured with MSCs pretreated with hypoxic TC supernatant (MSCs), Tregs co-cultured with CXCL5-knockdown MSCs pretreated with hypoxic TC supernatant (MSCs^CXCL5−^), Tregs co-cultured with CXCL6-knockdown MSCs pretreated with hypoxic TC supernatant (MSCs^CXCL6−^), Tregs co-cultured with CXCL5/6-double-knockdown MSCs pretreated with hypoxic TC supernatant (MSCs^CXCL5/6−^), CXCR1-knockdown Tregs co-cultured with MSCs pretreated with hypoxic TC supernatant (Treg^CXCR1−^). All groups were tested at varying Treg: CD8⁺ T cell ratios (1:1 to 1:32). *N* = 6. *p < 0.05, **p < 0.01, ***p < 0.001, ****p < 0.0001.** C** Flow cytometry analysis of CD25, FOXP3, and CXCR1 expression in Tregs under different treatment conditions. Treg, Tregs cultured alone; MSCs, Tregs co-cultured with MSCs pretreated with hypoxic TC supernatant; MSCs^CXCL5−^, Tregs co-cultured with CXCL5-knockdown MSCs pretreated with hypoxic TC supernatant; MSCs^CXCL6−^, Tregs co-cultured with CXCL6-knockdown MSCs pretreated with hypoxic TC supernatant; MSCs^CXCL5/6−^, Tregs co-cultured with CXCL5/6-double-knockdown MSCs pretreated with hypoxic TC supernatant. *N* = 6. *p < 0.05, **p < 0.01, ***p < 0.001, ****p < 0.0001. **D** Flow cytometry comparison of CD25, FOXP3, and CXCR1 expression among Tregs cultured alone (Treg), Tregs co-cultured with MSCs pretreated with hypoxic TC supernatant (MSCs), and CXCR1-knockdown Tregs (Treg^CXCR1−^). *p < 0.05, **p < 0.01, ***p < 0.001, ****p < 0.0001

To elucidate the role of CXCL5/6-CXCR1 axis in Treg recruitment, we employed siRNA-mediated knockdown of CXCL5/6 in MSCs or CXCR1 in Tregs, followed by Transwell migration assays. As shown in Fig. 5A, Treg migration was significantly enhanced in ^TC^MSC group compared to controls (*p* < 0.0001). Additionally, all subsequent references to “^TC^MSC” specifically denoted MSCs that were preconditioned with 5% O₂ TC-derived supernatant. This effect was abolished when CXCL5/6 were knocked down in MSCs (^TC^MSC^CXCL5/6−^) group, showing reduced migration (*p* < 0.001). Similarly, CXCR1-deficient Tregs (Tregs^CXCR1−^ group) exhibited impaired migration even toward hypoxic TC supernatant-conditioned MSCs (*p* < 0.001). Notably, CXCR1 re-expression in knockdown Tregs (Tregs^CXCR1− TC^MSC+CXCR1 group) restored migration capacity, confirming CXCR1’s determinative role. We further evaluated the axis’ influence on Treg immunosuppressive function. At varying Treg: CD8⁺ T cell ratios (1:1–1:32), Tregs co-cultured with MSCs pre-conditioned with hypoxic TC supernatant showed maximal suppression of CD8⁺ T cell cytotoxicity. This enhancement was attenuated when CXCL5/6 were knocked down in MSCs, with the most pronounced reduction observed in CXCL5/6-double-knockdown groups, suggesting synergistic effects. CXCR1-deficient Tregs displayed severely compromised suppression, failing to match control efficacy even at 1:1 ratio (Fig. 5B). Flow cytometry revealed that Tregs in ^TC^MSC group upregulated CD25, FOXP3, and CXCR1 versus controls, an effect diminished by CXCL5/6 knockdown (Fig. 5C). Correspondingly, Fig. 5D demonstrated that CXCR1 deficiency not only impaired migration but also reduced CD25 or FOXP3 expression.

### Hypoxic TC supernatant-conditioned MSCs improve survival and attenuate lung injury in humanized ALI mice

**Fig. 6:**
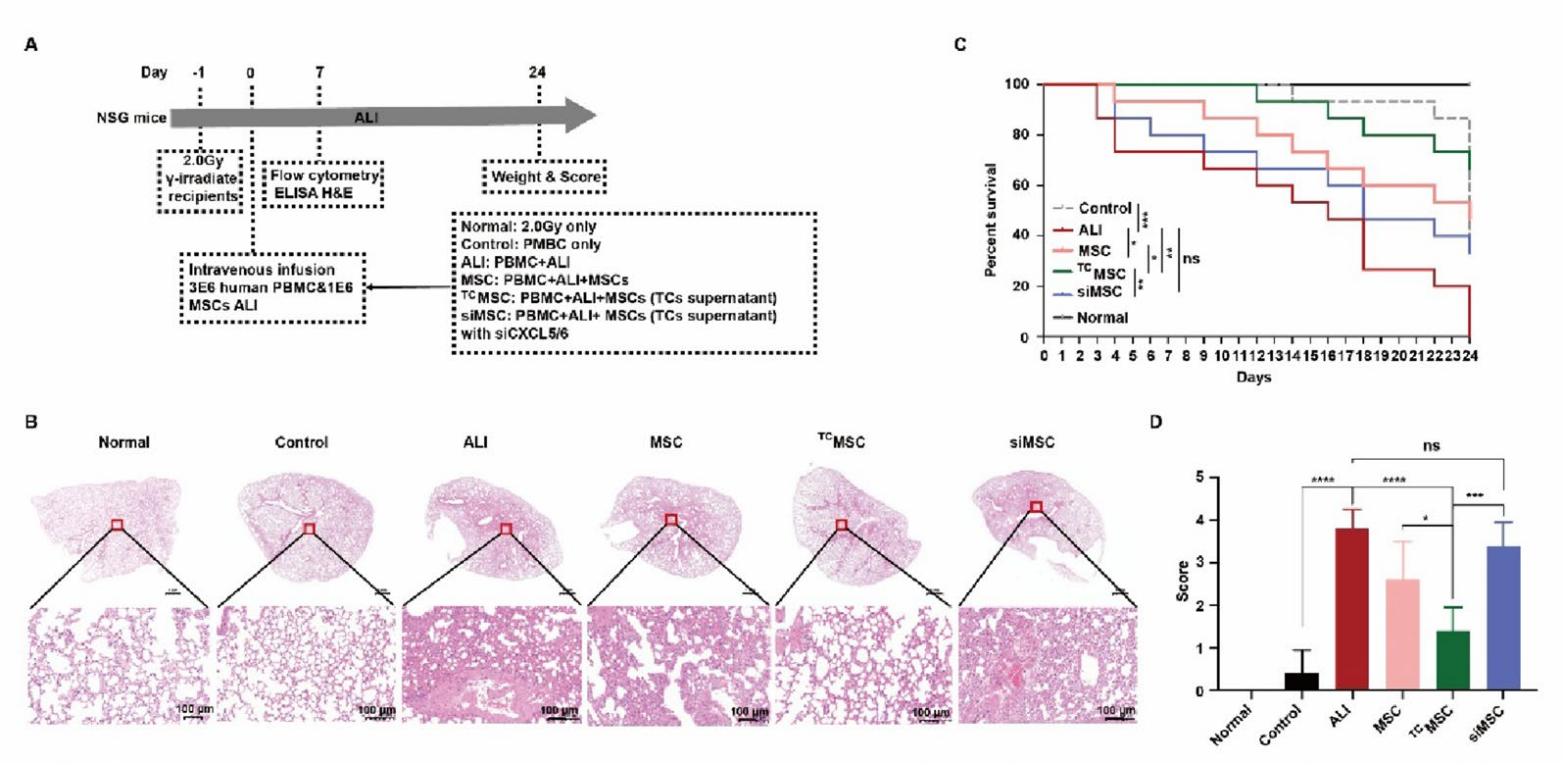
Therapeutic effects of hypoxic TC supernatant-conditioned MSCs in humanized ALI mice. ** A** Experimental design utilizing NSG mice subjected to 2.0 Gy γ-irradiation followed by intravenous administration of human peripheral blood mononuclear cells (PBMCs, 3 × 10⁶ cells) combined with interleukin-6 (IL-6) to establish ALI, with different experimental groups: irradiation-only controls, PBMC controls, ALI mice (PBMCs with ALI induction), MSC-treated ALI mice (PBMCs with ALI receiving MSCs), ^TC^MSC-treated ALI mice (PBMCs with ALI receiving TC supernatant-preconditioned MSCs), and siMSC-treated ALI mice (PBMCs with ALI receiving CXCL5/6-knockdown MSCs preconditioned with TC supernatant). Longitudinal assessment included flow cytometry, enzyme-linked immunosorbent assay (ELISA), hematoxylin and eosin (H&E) staining, and clinical scoring through day 24. ** B** Representative H&E-stained lung tissue sections. Scale bar = 200 μm. ** C** Survival analysis via Kaplan-Meier curves with mortality defined as ≥ 20% body weight loss. ** D** Quantitative assessment of lung injury scores. Normal, irradiation-only mice; Control, PBMCs-only mice; ALI, PBMCs-only ALI mice; MSC, ALI mice treated with PBMCs and MSCs; ^TC^MSC, ALI mice treated with PBMCs and TC supernatant-preconditioned MSCs; siMSC, ALI mice treated with PBMCs and CXCL5/6-knockdown MSCs preconditioned with TC supernatant. *p < 0.05, **p < 0.01, ***p < 0.001, ****p < 0.0001. ns, not significant

We established a humanized ALI model through γ-irradiation (2.0 Gy) and subsequent administration of human PBMCs with IL-6 in NSG mice, which recapitulated key pathological features of human ALI while enabling evaluation of therapeutic efficacy for hypoxic TC supernatant-conditioned MSCs (Fig. 6A). Analysis revealed severe inflammatory infiltration, alveolar wall thickening, and structural destruction in ALI mice in comparison with normal or control groups (Fig. 6B). While MSC monotherapy provided moderate histological improvement, persistent tissue damage remained. In contrast, TC supernatant-conditioned MSCs (^TC^MSC group) demonstrated significantly preserved alveolar architecture and reduced inflammation. Notably, CXCL5/6-knockdown MSCs preconditioned with TC supernatant (siMSC group) showed diminished therapeutic effects versus ^TC^MSC group (*p* < 0.01), underscoring the CXCL5/6-CXCR1 axis’s pivotal role. Survival analysis demonstrated that TC supernatant-conditioned MSC treatment significantly improved survival compared to conventional MSC therapy, while disruption of the CXCL5/6-CXCR1 axis markedly attenuated this protective effect (Fig. 6C). Quantitative assessment of lung injury severity demonstrated a progressive reduction in pathological scores across treatment groups, with TC supernatant-conditioned MSCs exhibiting the most pronounced protective effects (Fig. 6D). Notably, genetic disruption of the CXCL5/6-CXCR1 axis significantly compromised this therapeutic benefit, further validating the critical role of this signaling pathway in mediating lung protection.

**Fig. 7:**
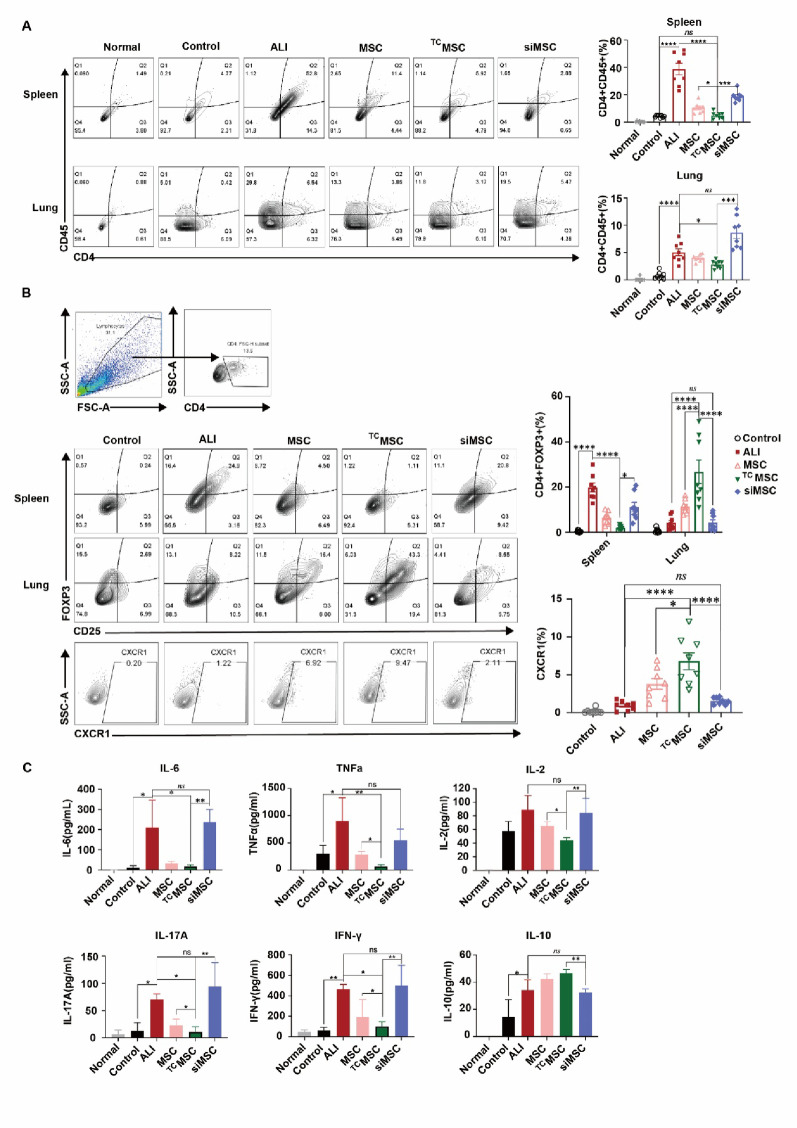
CXCL5/6 knockdown attenuated the therapeutic benefits of TC supernatant-preconditioned MSCs in humanized ALI mice. **A** Flow cytometric analysis of CD4^+^CD45^+^ effector T cell distribution in spleen and lung tissues across different treatment groups. *N* = 8. *p < 0.05, ***p < 0.001, ****p < 0.0001. ns, not significant. **B** Flow cytometry revealed significant alterations in pulmonary Treg proportion and their CXCR1 expression across treatment groups. *N* = 8. *p < 0.05, ****p < 0.0001. ns, not significant.** C** Comparative analysis of lung tissue homogenate cytokine profiles demonstrated differential expression of pro-inflammatory mediators ((IL-6, TNF-α, IL-17 A, IFN-γ), IL-2 and anti-inflammatory IL-10 across treatment groups. *N* = 8. *p < 0.05, **p < 0.01. ns, not significant. Normal, irradiation-only mice; Control, PBMCs-only mice; ALI, PBMCs-only ALI mice; MSC, ALI mice treated with PBMCs and MSCs; ^TC^MSC, ALI mice treated with PBMCs and TC supernatant-preconditioned MSCs; siMSC, ALI mice treated with PBMCs and CXCL5/6-knockdown MSCs preconditioned with TC supernatant

Flow cytometric analysis revealed differential CD4^+^CD45^+^ effector T cell profiles across treatment groups, with this pro-inflammatory cell population showing significantly higher proportion in ALI lung and spleen tissues compared to controls (*p* < 0.0001). Therapeutic ^TC^MSC administration markedly reduced pulmonary CD4^+^CD45^+^ T cell infiltration, whereas this anti-inflammatory effect was substantially reversed in the siMSC group (CXCL5/6-knockdown MSCs preconditioned with TC supernatant), leading to a significant rebound in effector T cell proportions (Fig. 7A). Flow cytometry revealed ^TC^MSC treatment significantly increased CXCR1 expression on pulmonary Tregs compared to other groups (*p* < 0.05, *p* < 0.0001), an effect that was abolished by CXCL5/6 knockdown in siMSC-treated ALI mice (*p* < 0.0001) (Fig. 7B). During ALI, while CD25^+^FOXP3^+^ Tregs were significantly elevated in the spleen but remained low in the lungs, ^TC^MSC treatment reversed this distribution by promoting greater Treg accumulation in the lungs compared to the spleen, suggesting ^TC^MSC-mediated recruitment of Tregs from peripheral circulation to the injured lung tissue. This effect was significantly attenuated upon CXCL5/6 interference (*p* < 0.001), indicating these chemokines’ critical role in Treg recruitment. Lung tissue cytokine profiling revealed that ^TC^MSC treatment significantly reduced pro-inflammatory mediators (IL-6, TNF-α, IL-17 A, IFN-γ) along with IL-2 (*p* < 0.05, *p* < 0.01) while elevating anti-inflammatory IL-10 (*p* < 0.05), whereas the siMSC group maintained elevated pro-inflammatory cytokines and reduced IL-10 levels (Fig. 7C).

## Discussion

This study demonstrated that MSCs pre-conditioned with supernatant from TCs cultured under 5% hypoxic condition significantly ameliorated lung injury through the enhanced recruitment and immunosuppressive function of Tregs. Our findings revealed a potential mechanism by which TCs-derived factors may augment MSC-mediated immunomodulation via the CXCL5/6-CXCR1 axis, contributing to the optimization of cellular therapies for ALI.

The use of TC supernatant-preconditioned MSCs was found to offer improved efficacy compared to monotherapy strategies in our experimental system. While previous studies had reported modest efficacy of MSC monotherapy in ALI/ARDS models [14, 15], our findings indicated that MSCs pre-conditioned with hypoxic TC supernatant showed improved therapeutic outcomes characterized by preserved alveolar architecture, reduced inflammatory infiltration, and decreased pro-inflammatory cytokine profiles. This aligned with emerging evidence suggesting that optimizing cellular therapy efficacy necessitates comprehensive integration of microenvironmental context and intercellular signaling networks [16–18].

The CXCL5/6-CXCR1 chemokine axis identified in our study represented a distinct mechanistic pathway underlying enhanced Treg recruitment to inflamed lung tissues. Hypoxia-cultured TCs were found to significantly upregulated MSC secretion of CXCL5/6, which in turn promoted CXCR1 expression on Tregs, facilitating their targeted migration to inflammatory sites. Importantly, this upregulation of CXCL5/6 occurred selectively while most other inflammatory chemokines showed no significant increase or were downregulated, and Tregs exhibited marked enrichment of chemokine receptor signaling pathways in transcriptomic analyses. This finding is consistent with recent studies highlighting the importance of precise control of immune cell trafficking in resolving tissue inflammation [19, 20]. Moreover, our in vivo experiments using siRNA-mediated knockdown confirmed the indispensable role of this axis, as disruption of either CXCL5/6 in MSCs or CXCR1 in Tregs significantly attenuated therapeutic efficacy. These results collectively provide strong rationale for further investigation of this specific chemokine axis in Treg recruitment and inflammatory resolution.

Beyond enhancing Treg recruitment, our data indicated that the TC-conditioned MSC approach appeared to modulate Treg phenotype and function. Tregs co-cultured with TC supernatant-preconditioned MSCs exhibited significantly elevated expression of key immunoregulatory molecules (FOXP3, CTLA4, PD1) and enhanced suppressive capacity against effector T cells. Notably, FOXP3—the master transcriptional regulator of Treg stability and function—showed sustained high expression, suggesting robust Treg functional integrity [21]. Furthermore, these Tregs secreted elevated levels of anti-inflammatory cytokines (e.g., IL-10, TGF-β), reinforcing their immunosuppressive potential. Mechanistically, Fig. 4B linked this functional modulation to CXCL5/6-CXCR1 and CSF signaling, as well as IL-33-ST2 axis activation—the latter being critical for both Treg chemotaxis toward inflammatory sites and their functional reinforcement [22], underscoring the immunoregulatory synergy driven by TC-conditioned MSCs. This functional enhancement appeared to be mechanistically linked to the CXCL5/6-CXCR1 axis, as disruption of this pathway not only impaired migration but also compromised suppressive function. These findings were consistent with emerging concepts of “trained immunity” wherein innate immune responses can be functionally reprogrammed through specific microenvironmental cues [23, 24].

The humanized ALI mouse model employed in our study provided an experimental platform to evaluate human cell therapeutic efficacy in a clinically relevant context. This approach helps bridge translational challenges in preclinical ALI studies, where conventional murine models often fail to fully replicate key immunopathological features of human disease [25–27]. Our platform enabled direct evaluation of TC-conditioned human MSCs’ immunomodulatory effects on human immune cells within a living system - a technically challenging but scientifically valuable achievement. The engrafted human immune cells selectively induced pulmonary inflammation without significant extra-pulmonary involvement (Fig. S5), creating an ideal scenario to validate our in vitro findings. Importantly, TC-conditioned MSCs significantly improved survival and attenuated lung injury severity in this humanized model, demonstrating consistent immunosuppressive capacity by significantly reducing CD4^+^CD45^+^ T cell infiltration in both circulation and lung tissue while enhancing pulmonary Treg recruitment and anti-inflammatory cytokine profiles.

This study demonstrated that hypoxia-cultured TC supernatant enhanced MSC therapeutic efficacy in ALI by modulating the CXCL5/6-CXCR1 axis while simultaneously improving MSC properties through reduced inflammatory factor secretion and enhanced proliferation capacity. These findings suggested that TCs could function as a novel biological catalyst for optimizing MSC-based therapies. The data provided new insights for developing next-generation MSC therapeutics capable of concurrently addressing disease pathology and improving cellular performance. Although certain limitations were identified, including the need for clinical dosing optimization and potential involvement of additional molecular pathways, our results established TC-conditioned MSCs as a promising strategy to advance stem cell therapy applications.

## Conclusion

In summary, our study demonstrated that MSCs pre-conditioned with hypoxic TC supernatant significantly ameliorates LPS-induced lung injury through enhanced Treg recruitment and function mediated by the CXCL5/6-CXCR1 axis. These findings offer preliminary evidence supporting a mechanistic framework for optimizing cellular therapy for ALI and potentially other inflammatory disorders characterized by immune dysregulation. Future studies would need to focus on refining this therapeutic approach for clinical translation, including optimization of cell preparation protocols, determination of optimal administration parameters, and evaluation in diverse ALI patient populations.

## Supplementary Information

Below is the link to the electronic supplementary material.

Supplementary material 1.

Supplementary material 2.

## Data Availability

The original research data presented in this study are included in the article and Supplementary Material. Raw data have been deposited to National Center for Biotechnology Information (NCBI) under the BioProject number PRJNA1273917. Further inquiries can be directed to the corresponding author.
