# Supplementary material for: Hypoxic TCs-preconditioned MSCs ameliorate acute lung injury via enhanced Treg recruitment and function through CXCL5/6-CXCR1 axis
Source: Stem Cell Res Ther. 2025 Dec 26;17:54. doi: 10.1186/s13287-025-04858-6 (PMC12849632; doi:10.1186/s13287-025-04858-6)
Supplement: Supplementary file 1 — Supplementary material 1. [file 13287_2025_4858_MOESM1_ESM.docx]

**Hypoxic Telocyte-Preconditioned Mesenchymal Stem Cells Ameliorate Acute Lung Injury via Enhanced Regulatory T Cell Recruitment and Function Through the CXCL5/6-CXCR1 Axis**

Luoyue Yin, ^1,2^† Xu Zhang,^3,4^† Yile Zhou, ^1,2^† Huihui Ju,^1,2^ Youwei Zhu,^5^ Rongrong Gao,^6^* Pinwen Wu,^7^* Hao Fang ^1,2^*

^1^Department of Anaesthesiology, Zhongshan Hospital, Fudan University, 180 Fenglin Road, Shanghai 200032, China.

^2^Department of Anaesthesiology, Shanghai Geriatric Medical Center, Shanghai 201104, China.

^3^NHC Key Lab of Reproduction Regulation, Shanghai Engineering Research Center of Reproductive Health Drug and Devices, Shanghai Institute for Biomedical and Pharmaceutical Technologies, Shanghai 200237, China.

^4^Shanghai-MOST Key Laboratory of Health and Disease Genomics, NHC Key Lab of Reproduction Regulation, Shanghai Institute for Biomedical and Pharmaceutical Technologies, Shanghai 200237, China.

^5^Clinical Center of Bio-Therapy at Zhongshan Hospital & Institutes of Biomedical Sciences, Shanghai Public Health Clinical Center, Fudan University, Shanghai, China.

^6^Clinical Center for Biotherapy at Zhongshan Hospital, Fudan University, Shanghai 200032, China.

^7^Department of Anesthesiology, Minhang Hospital, Fudan University, Shanghai, China.

†These authors contributed equally to this work

Correspondence: Hao Fang [drfanghao@163.com](mailto:drfanghao@163.com)

Co-correspondence: Rongrong lsq274241075@163.com; Gao Pinwen Wu [wpw_1976@163.com](mailto:wpw_1976@163.com)

**Supplementary Materials**

**Materials and Methods**

**Screening and selection of siRNA sequences**

Three different siRNA sequences targeting each gene of interest (CXCL5 and CXCL6) were obtained from Bioscien. To identify the most effective siRNA sequences, GFP reporter plasmids containing the target gene sequences were constructed and amplified. HEK293T cells were co-transfected with these GFP reporter plasmids and the corresponding siRNAs at plasmid : siRNA ratios of 1:1 and 1:2 using Lipofectamine 3000 (Invitrogen, #L3000015). At 48 hours post-transfection, knockdown efficiency was evaluated by fluorescence microscopy and flow cytometric analysis of GFP intensity. The siRNA sequences demonstrating the highest knockdown efficiency were selected for subsequent experiments.

The siRNA sequences used were as follows:

CXCL5 sequence 1: 5′-UGAAUUGUAGGUGACUAUUAUtt-3′

CXCL5 sequence 2: 5′-GCGUUUGUUUACAGACCACGCtt-3′

CXCL5 sequence 3: 5′-GUAAUCUGCAAGUGUUCGCCAtt-3′

CXCL6 sequence 1: 5′-UUACGCUGAGAGUAAACCCCAtt-3′

CXCL6 sequence 2: 5′-UUGUCUGGACCCGGAAGCCCCtt-3′

CXCL6 sequence 3: 5′-UAGUCUUUCAAUGAAUAUUGAtt-3′

**siRNA-mediated gene knockdown of Tregs**

Tregs were electroporated with siRNA using the Celetrix^TM^EX+ system (Supplementary Figure 4). Briefly, 4×10^7^ cells were mixed with 50 μg siRNA and pulsed at 1300 V in a 4-mm cuvette, followed by recovery in complete medium at 37°C. siRNA sequence were as follows：

siCXCR1-1：CCAAGACAAACAAACUUGACCtc

UUCUUCGUCUGUCAAUGUCUtt

siCXCR1-2:AGACAUUGACAGACGAAGAAGtt

UGCAGAUGAAGAUUACAGCCtt

siCXCR1-3:GGOUGUAAUCUUCAUCUGCAGtt

UGGACCACCUGGACAAGCAAAtt

**Culture and siRNA-mediated gene knockdown of MSCs**

Human MSCs were obtained from Hycells (#MSC331) and recovered from cryopreservation in liquid nitrogen. Cells were cultured in Mesenchymal Stem Cell Basal Medium (MSCBM; Dakewe Biotech, #6114011) supplemented with growth factors according to the manufacturer's recommendations. Cultures were maintained at 37°C in a humidified atmosphere containing 5% CO_2_. Cell passaging was performed using 0.25% Trypsin-EDTA solution (Gibco, #25200056) when cultures reached 80-90% confluence.

For CXCL5 and CXCL6 knockdown experiments (Supplementary Figures 2 and 3), MSCs were seeded 18-24 hours prior to transfection to achieve 30-50% confluence. Transfection was performed using CALNP™ RNAi in vitro transfection reagent (D-Nano Therapeutics) according to the manufacturer's protocol. Small interfering RNAs (siRNAs) targeting CXCL5 and CXCL6 (Bioscien) were used to induce gene silencing.

**Cell lines and culture conditions**

Human embryonic kidney cells (HEK293T) (ATCC #CRL-3216) were maintained in DMEM (Corning, #10-013-CV). Lung cancer cells (NCI-H292) (ATCC #CRL-1848) were cultured in RPMI 1640 medium (Corning, #10-040-CVR). Both culture media were supplemented with 10% FBS (Gibco, #10099-141) and 1% penicillin-streptomycin solution (Servicebio, CR2111115). All cell lines were maintained at 37°C in a humidified atmosphere containing 5% CO_2_ and regularly tested for mycoplasma contamination.

**Flow cytometry analysis**

Cell surface marker staining was performed in the dark using fluorochrome-conjugated antibodies diluted in FACS buffer (PBS containing 2% FBS). Zombie Aqua™ viability dye (BioLegend, #423101) was included to discriminate live from dead cells. Staining was conducted for 20 minutes at room temperature or 30 minutes at 4°C. Following incubation, cells were washed twice with FACS buffer and either resuspended in 200 μL FACS buffer for immediate analysis or processed for subsequent intracellular staining. Following surface marker and viability staining, cells were washed twice with FACS buffer. Fixation and permeabilization were performed using the Foxp3/Transcription Factor Staining Buffer Set (eBioscience, #00-5521-00) according to the manufacturer's protocol. Intracellular staining was then performed using Foxp3-BV421 antibody (BioLegend, #320124) at the recommended dilution.

The following antibodies were used in this study: Foxp3-BV421 (BioLegend, #320124), CD25-FITC (Elabscience, #E-AB-F1194C), CD45-PE (Elabscience, #E-AB-F1137D), CD4-APC (Elabscience, #E-AB-F1109E), Zombie Aqua™ viability dye (BioLegend, #423101), and cell proliferation dye eFluor670 (Invitrogen, #00-5523).

Flow cytometry data acquisition was performed using BD FACS Aria III and BD FACS Aria II instruments (BD Biosciences). Cell sorting was conducted using a BD FACS Aria III Cell Sorter (BD Biosciences). Flow cytometry data were analyzed using FlowJo software version 10 (Tree Star, Inc.).

**RNA Sequencing and Bioinformatic Analysis**

Tregs were sorted based on specific surface markers, and total RNA was extracted using TRIzol reagent (Invitrogen) according to the manufacturer's instructions. Extracted RNA was stored at -80°C until further processing. RNA quality and integrity were assessed using the Agilent 2100 Bioanalyzer (Agilent Technologies).

Sequencing libraries were prepared using EASY RNA-SEQ technology. To normalize gene expression data for differences in sequencing depth and gene length, fragments per kilobase of exon model per million mapped fragments (FPKM) values were calculated. Differential gene expression analysis, heatmap generation, and Kyoto Encyclopedia of Genes and Genomes (KEGG) pathway analyses were performed using Sangerbox Online software (http://vip.sangerbox.com/). Gene set enrichment analysis (GSEA) was conducted using log2 RPKM data to determine whether specific gene signatures were significantly enriched in particular cell subsets. One thousand random permutations of phenotypic subgroups were used to establish null distribution of enrichment scores, against which normalized enrichment scores (NES) and false discovery rate (FDR)-corrected q-values were calculated.

**Lung injury assessment**

For LPS mice model, lung injury was assessed using the Smith scoring method, which evaluates pulmonary edema, alveolar and interstitial inflammation, alveolar and interstitial hemorrhage, atelectasis, and hyaline membrane formation. Each parameter was scored on a scale of 0-4: no injury (0), lesion affecting <25% of field (1), lesion affecting 25-50% of field (2), lesion affecting 50-75% of field (3), and lesion affecting >75% of field (4). The total lung injury score was calculated as the sum of individual parameter scores. Ten high-magnification fields were evaluated for each animal, and the average score was determined.

| **A Four-Level Grading System** | | |
| --- | --- | --- |
| Level（number） | Category | Introduction |
| 0 | No injuries | The lung tissue is normal. |
| 1 | Mild | Area of damage＜25% |
| 2 | Moderate | 25%＜Area of damage＜50% |
| 3 | Severe | 50%＜Area of damage＜75% |
| 4 | Serious | Area of damage＞75% |

**Animal experiments**

All animal experiments were approved by the Animal Ethics Committee of Zhongshan Hospital, Fudan University. All experimental procedures were performed in strict accordance with the Guidelines established by Zhongshan Hospital, Fudan University.

Female NOD.Cg*-*Prkdc^scid^Il2rg^tm1Sug^/JicCrl (NOG) mice (6-8 weeks old) were purchased from Biocytogen Co Ltd and housed under specific pathogen-free (SPF) conditions at the animal facilities of Shanghai Branch of Chinese Academy of Science (Shanghai, China). Male C57BL/6 mice (6-8 weeks old) were purchased from Biocytogen Co Ltd and housed under SPF conditions at the Laboratory Animal Center of Fudan University (Shanghai, China). The mice were maintained in individually ventilated cages with controlled temperature (22±1°C), humidity (50±5%), and a 12-hour light/dark cycle, provided with autoclaved corn cob bedding, irradiated food and sterile water. Animals were randomly allocated to control and treatment groups using a computer-generated random number sequence (Excel RAND function). Cage positions were rotated daily to minimize environmental confounding, and researchers were blinded to group assignments during data collection.

For anesthesia, mice received intraperitoneal injection of sodium pentobarbital (50 mg/kg, Sigma-Aldrich, Cat# P3761) dissolved in sterile saline (10 mg/mL). Depth of anesthesia was verified by absence of toe-pinch reflex prior to procedures. Following deep anesthesia, cardiac puncture was performed for terminal tissue collection. Mice were then transcardially perfused with PBS (pH 7.4, Gibco™, Cat# 10010023) prior to tissue harvesting.

NOG mice were subjected to 2.0 Gy of total body irradiation one day prior to cell injection. Human PBMCs (3×10^6^ cells per mouse, n = 8 per group) and differentially treated MSCs (1×10^6^ cells per mouse, n = 8 per group) were injected intravenously. Samples were collected on days 7 and 24 post-injection as described in subsequent sections.

Postoperative monitoring included assessment of body weight, activity levels, and respiratory status. Animals meeting endpoint criteria were euthanized by pentobarbital overdose followed by cervical dislocation. No unanticipated adverse events were observed during the study period.

**Sample collection and processing**

Body weight was measured on the day of model induction and daily thereafter until day 24. Blood was collected after anesthesia by retro-orbital bleeding. Serum was separated by centrifugation at 3000 rpm for 15 minutes and used for enzyme-linked immunosorbent assay (ELISA). Plasma, after erythrocyte lysis, was combined with spleen cells for flow cytometric analysis. Lung tissue was divided into three portions: one portion was enzymatically digested to obtain single-cell suspensions for flow cytometry, another was fixed in 4% formaldehyde for paraffin embedding and histopathological examination, and the third portion was homogenized for ELISA (Elabscience, #E-EL-H0046, #E-EL-H0047). Spleen was mechanically disaggregated to generate single-cell suspensions for flow cytometry. After erythrocyte lysis (Invitrogen, #00-4300-54), cells were stained with human-specific antibodies and analyzed by flow cytometry.

**Immunohistochemistry**

Organs were fixed in 4% paraformaldehyde for 24 hours, washed, and transferred to 75% ethanol prior to tissue processing. Paraffin-embedded tissues were sectioned at 5 μm thickness and stained with H&E to evaluate inflammatory infiltration. For quantitative analysis, stained slides were digitally scanned using TissueFAXS 200 (TissueGnostics).

**
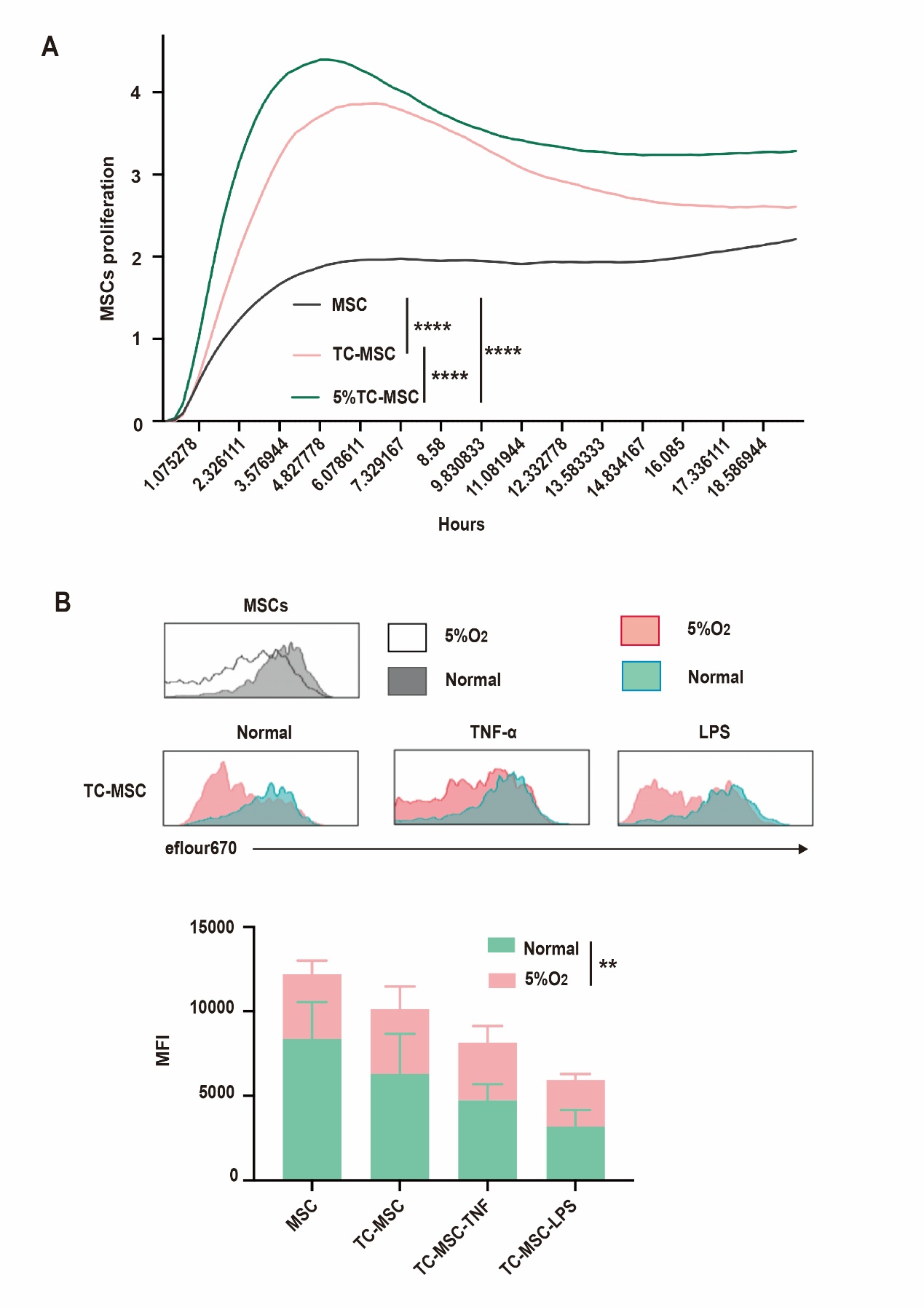
**

**Figure S1.** Effects of TCs-derived supernatant on MSC proliferation. A) RTCA showing proliferation of MSCs (1×10⁴ cells/well) over time when treated with control medium, normoxic TCs-derived supernatant, or 5% hypoxic TCs-derived supernatant. **** *p* < 0.0001. B) Flow cytometry analysis of eFluor670-labeled MSCs with different treatments (control medium, TCs-derived supernatant, TNF-α-stimulated TCs-derived supernatant, or LPS-stimulated TCs-derived supernatant under normoxic or 5% hypoxic conditions. Upper panels showed representative flow cytometry histograms; lower panel shows MFI quantification. ** *p* < 0.01.


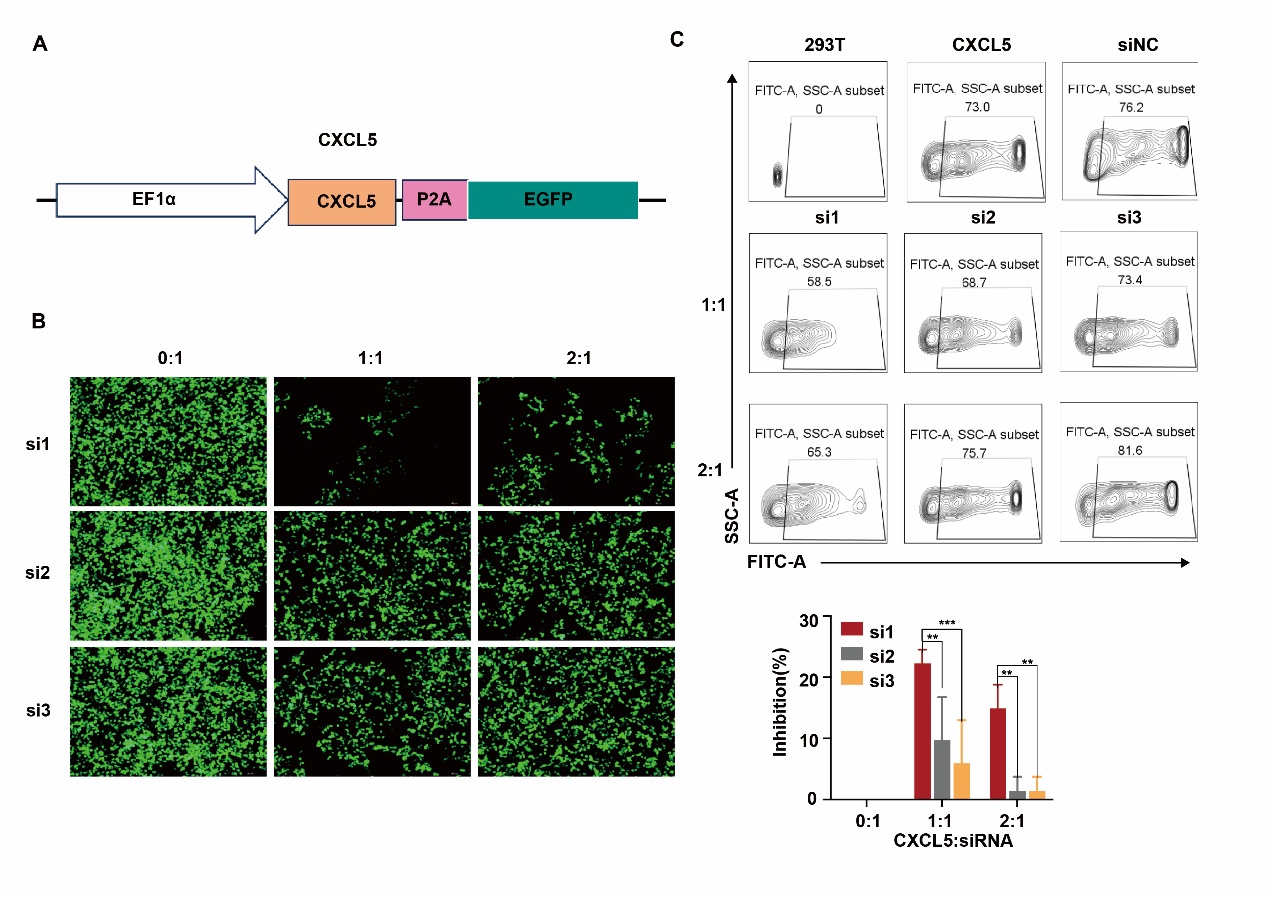


**Figure S2.** Evaluation of CXCL5 siRNA knockdown efficiency in 293T cells. A) Schematic representation of the CXCL5 overexpression construct containing EF1α promoter, CXCL5 coding sequence, P2A self-cleaving peptide, and EGFP reporter. B) Fluorescence microscopy images of 293T cells co-transfected with CXCL5 expression plasmid and three different siRNA sequences (si1, si2, si3) at molar ratios of 0:1, 1:1, and 2:1. Images were captured 48h post-transfection. C) Flow cytometric analysis of EGFP expression in transfected cells. Upper panels showed representative FITC-A/SSC-A plots with percentage of EGFP-positive cells from control groups (293T, CXCL5 overexpression, siNC) and experimental groups with varying ratios of siRNAs. Lower panel showed quantification of CXCL5 inhibition percentage across different conditions. ***p* < 0.01, ****p* < 0.001.


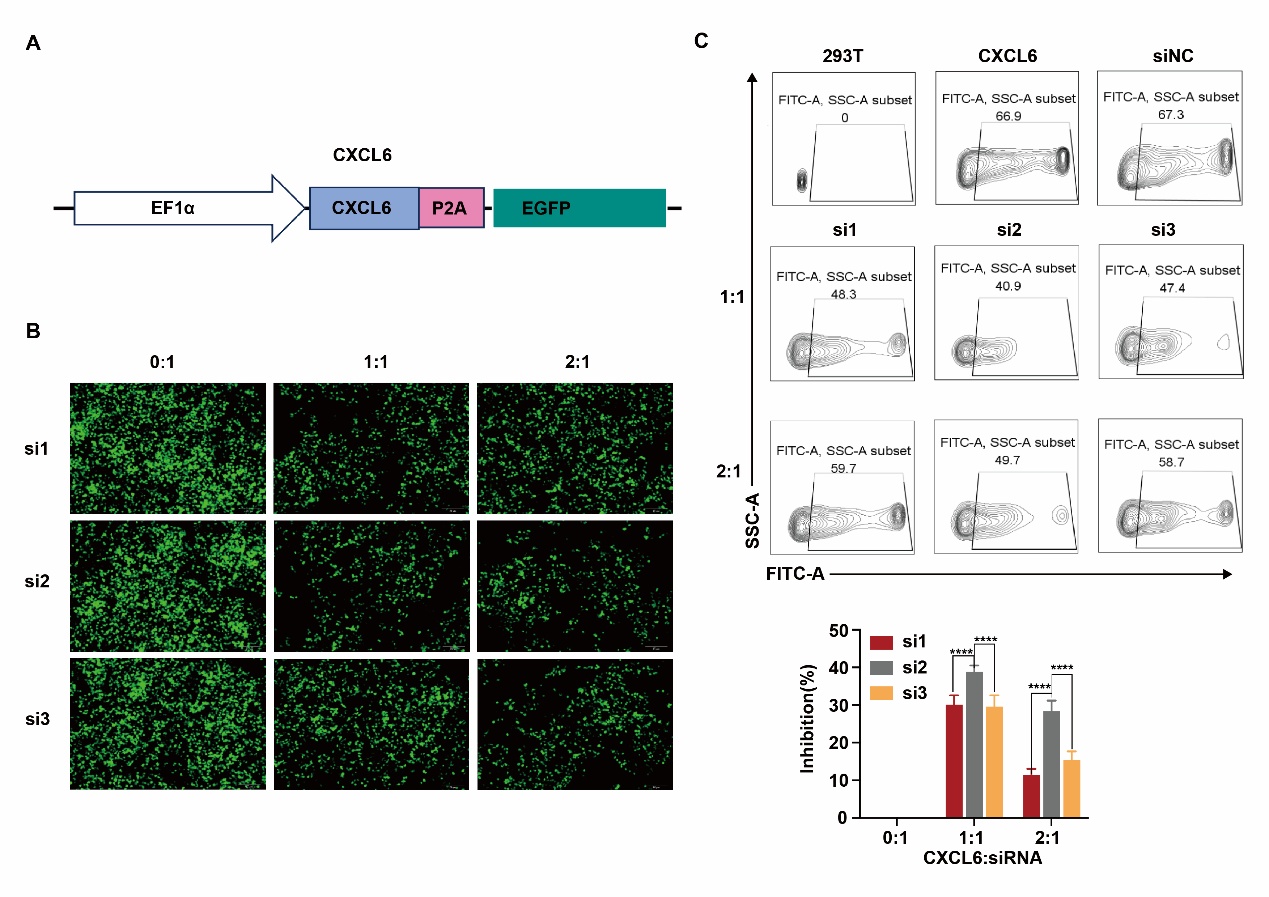


**Figure S3.** Assessment of CXCL6 siRNA knockdown efficiency in 293T cells. A) Schematic representation of the CXCL6 overexpression construct containing EF1α promoter, CXCL6 coding sequence, P2A self-cleaving peptide, and EGFP reporter. B) Fluorescence microscopy images of 293T cells co-transfected with CXCL6 expression plasmid and three different siRNA sequences (si1, si2, si3) at molar ratios of 0:1, 1:1, and 2:1. Images were captured 48h post-transfection. C) Flow cytometric analysis of EGFP expression in transfected cells. Upper panels show representative FITC-A/SSC-A plots with percentage of EGFP-positive cells from control groups (293T, CXCL6 overexpression, siNC) and experimental groups with varying ratios of siRNAs. Lower panel shows quantification of CXCL6 inhibition percentage across different conditions. *****p* < 0.0001.


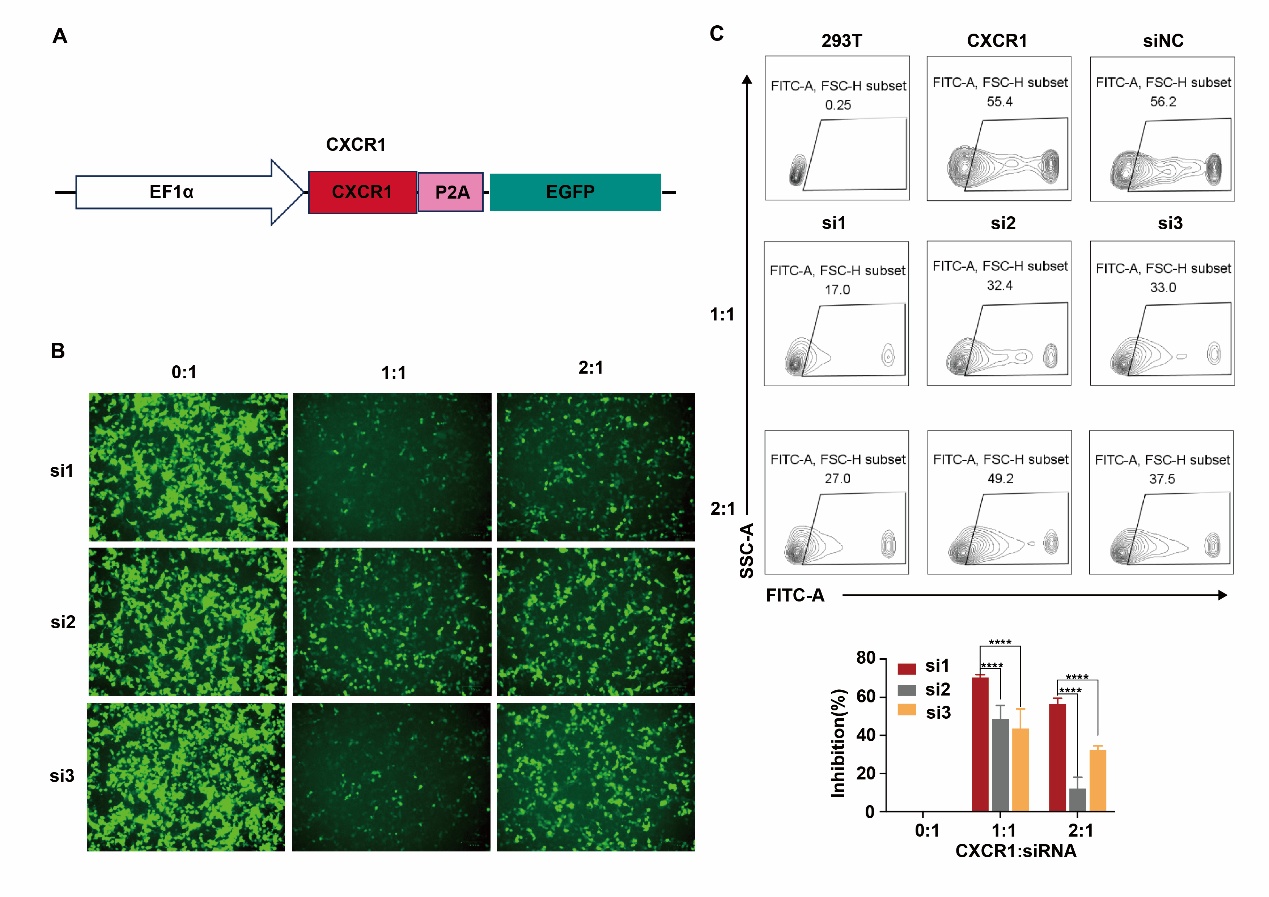


**Figure S4.** Evaluation of CXCR1 siRNA knockdown efficiency in 293T cells. A) Schematic representation of the CXCR1 overexpression construct containing EF1α promoter, CXCR1 coding sequence, P2A self-cleaving peptide, and EGFP reporter. B) Fluorescence microscopy images of 293T cells co-transfected with CXCR1 expression plasmid and three different siRNA sequences (si1, si2, si3) at molar ratios of 0:1, 1:1, and 2:1. Images were captured 48h post-transfection. C) Flow cytometric analysis of EGFP expression in transfected cells. Upper panels show representative FITC-A/FSC-H plots with percentage of EGFP-positive cells from control groups (293T, CXCR1 overexpression, siNC) and experimental groups with varying ratios of siRNAs. Lower panel shows quantification of CXCR1 inhibition percentage across different conditions. *****p* < 0.0001.

**
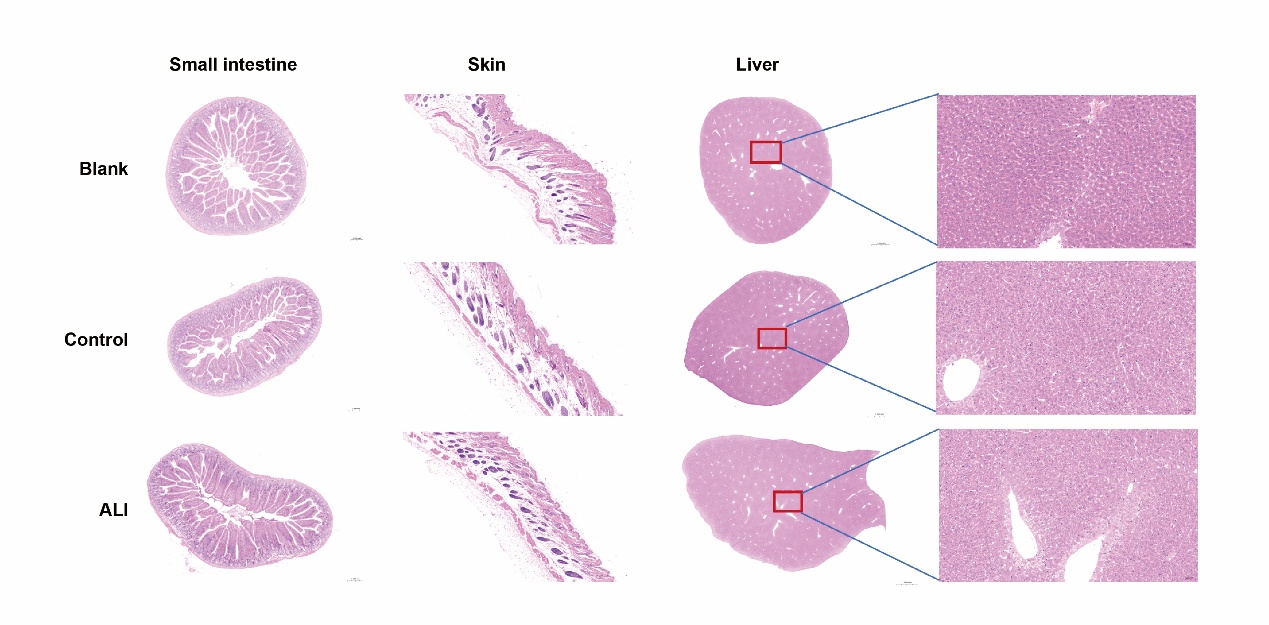
**

**Figure S5.** Histopathological assessment of potential GVHD in multiple organs of humanized mice with LPS-induced lung injury. Representative H&E staining of small intestine, skin, and liver tissues from different experimental groups one week after LPS-induced lung inflammation (when GVHD typically begins to manifest). Groups include: Blank (non-humanized control mice), Control (humanized mice without lung inflammation induction), and ALI (humanized mice with acute lung injury). Liver sections were shown at low magnification with high-magnification insets (red boxes). The results demonstrated that despite pulmonary inflammation in the ALI group, other GVHD-susceptible organs (small intestine, skin, and liver) showed no histological evidence of GVHD pathology, confirming the localized nature of the inflammatory response to the lungs.
