# Supplementary material for: Hypoxic TCs-preconditioned MSCs ameliorate acute lung injury via enhanced Treg recruitment and function through CXCL5/6-CXCR1 axis
Source: Stem Cell Res Ther. 2025 Dec 26;17:54. doi: 10.1186/s13287-025-04858-6 (PMC12849632; doi:10.1186/s13287-025-04858-6)
Supplement: Supplementary file 2 — Supplementary material 2. [file 13287_2025_4858_MOESM2_ESM.docx]

**
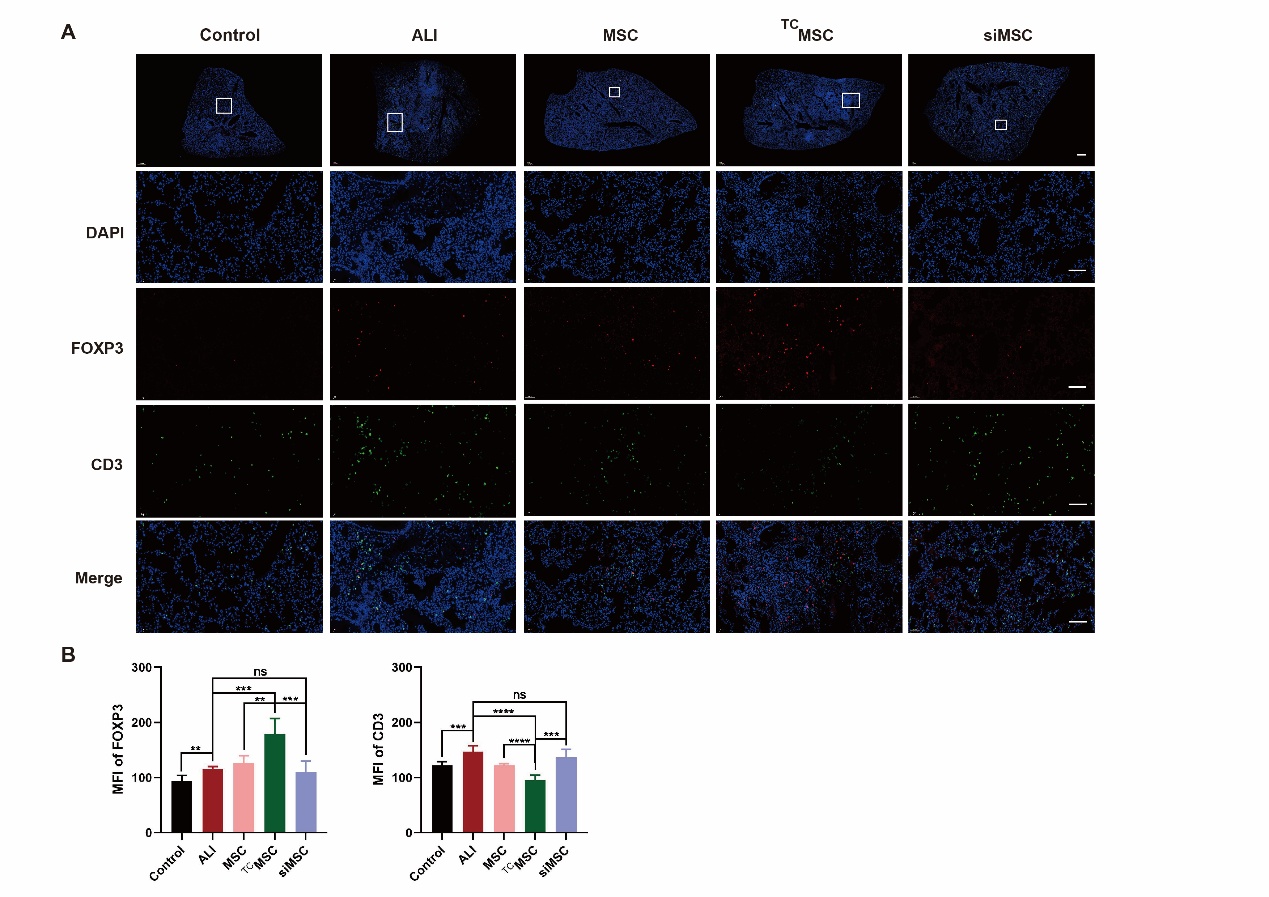
**

**Supplementary Fig. 1** Immunofluorescence analysis of FOXP3 and CD3 expression in lung tissue sections of humanized ALI mice under different treatments. (A) Representative immunofluorescence images of FOXP3 (red), CD3 (green) and nuclei (DAPI, blue). Scale bar = 500 µm; Scale bar = 100 µm. (B) Quantitative analysis of FOXP3 and CD3 fluorescence intensity. Control, PBMCs-only mice; ALI, PBMCs-only ALI mice; MSC, ALI mice treated with PBMCs and MSCs; ^TC^MSC, ALI mice treated with PBMCs and TC supernatant-preconditioned MSCs; siMSC, ALI mice treated with PBMCs and CXCL5/6-knockdown MSCs preconditioned with TC supernatant. ***p* < 0.01, ****p* < 0.001, *****p* < 0.0001. ns, not significant.


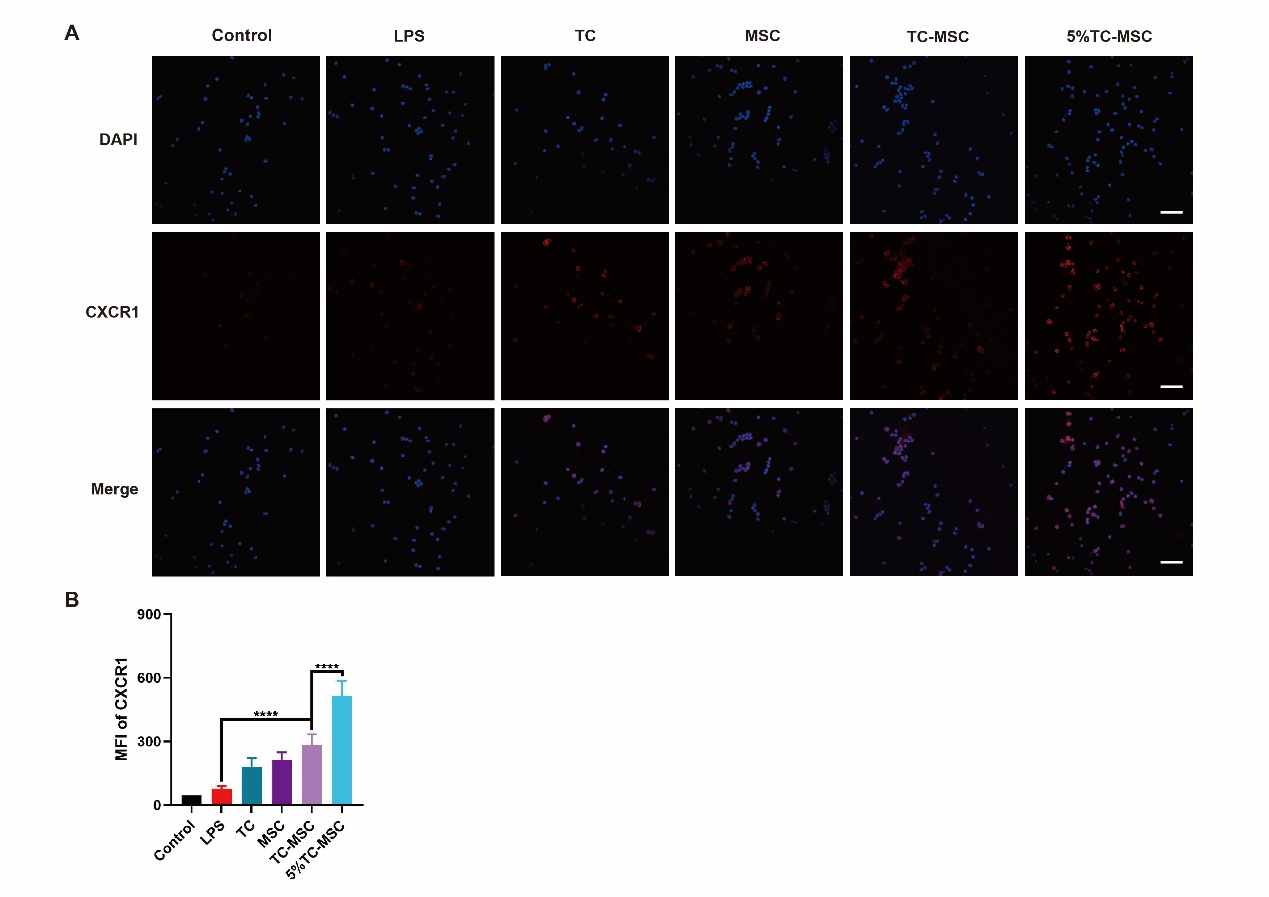


**Supplementary Fig. 2** Immunofluorescence analysis of CXCR1 expression in Tregs under different treatments. (A) Representative immunofluorescence images of CXCR1 (red) and nuclei (DAPI, blue). Scale bar = 100 µm. (B) Quantitative analysis of CXCR1 fluorescence intensity. Control, Tregs alone; LPS, Tregs stimulated with LPS; TC, Tregs co-cultured with TCs; MSC, Tregs co-cultured with MSCs; TC-MSC, Tregs co-cultured with MSCs pre-conditioned with normoxic TC-derived supernatant; 5% TC-MSC, Tregs co-cultured with MSCs pre-conditioned with supernatant from TCs cultured under 5% hypoxic conditions. N=6. *****p* < 0.0001.


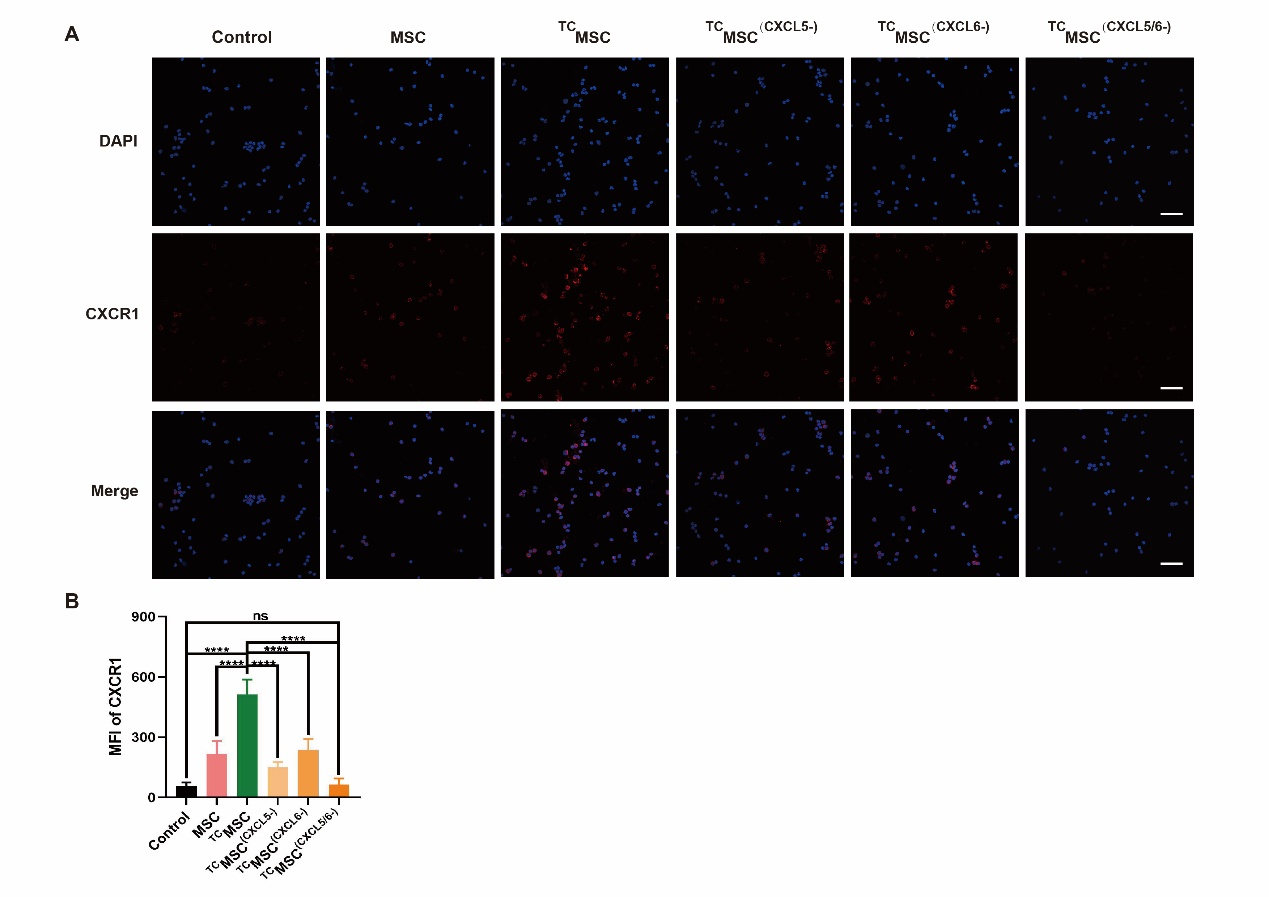


**Supplementary Fig. 3** Immunofluorescence analysis of CXCR1 expression in Tregs under different treatments. (A) Representative immunofluorescence images of CXCR1 (red) and nuclei (DAPI, blue). Scale bar = 100 µm. (B) Quantitative analysis of CXCR1 fluorescence intensity. Control, Tregs cultured alone; MSC, Tregs co-cultured with MSCs; ^TC^MSC, Tregs cocultured with MSCs pretreated with hypoxic TC supernatant; ^TC^MSC^(CXCL5-)^, Tregs cocultured with MSCs pretreated with hypoxic TC supernatant and underwent additional siRNA-mediated CXCL5 knockdown; ^TC^MSC^(CXCL6-)^, Tregs cocultured with MSCs pretreated with hypoxic TC supernatant and underwent additional siRNA-mediated CXCL6 knockdown; ^TC^MSC^(CXCL5/6-)^, Tregs cocultured with MSCs pretreated with hypoxic TC supernatant and underwent additional siRNA-mediated CXCL5/6 knockdown. N = 6. *****p* < 0.0001, ns, not significant.


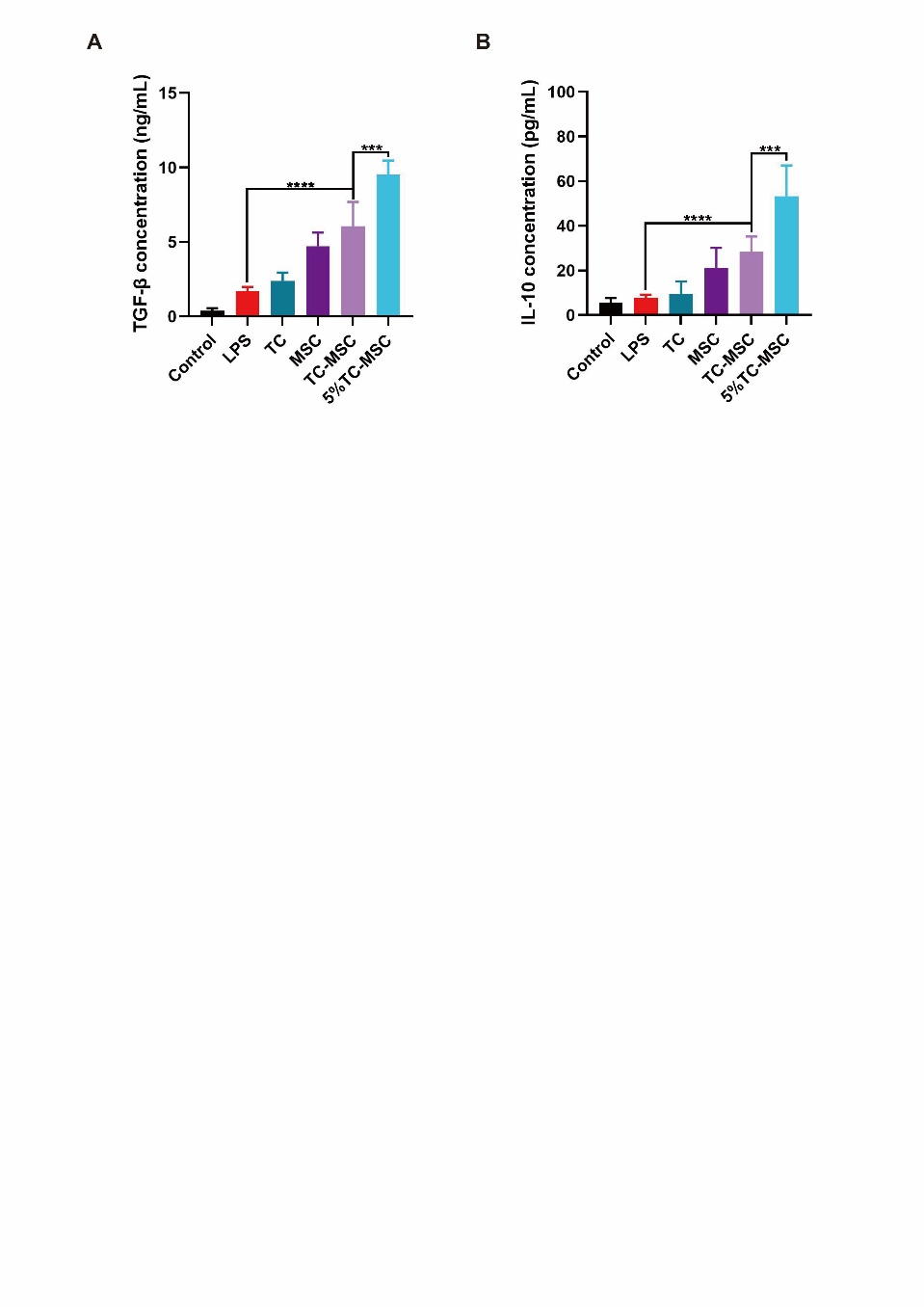


**Supplementary Fig.4** ELISA detection of TGF-β and IL-10 in the conditioned medium from Tregs under different treatments. (A) Concentration of TGF-β in the conditioned medium from Tregs. (B) Concentration of IL-10 in the conditioned medium from Tregs. Control, Tregs alone; LPS, Tregs stimulated with LPS; TC, Tregs co-cultured with TCs; MSC, Tregs co-cultured with MSCs; TC-MSC, Tregs co-cultured with MSCs pre-conditioned with normoxic TC-derived supernatant; 5% TC-MSC, Tregs co-cultured with MSCs pre-conditioned with supernatant from TCs cultured under 5% hypoxic conditions. N=6. ****p* < 0.001, *****p* < 0.0001.


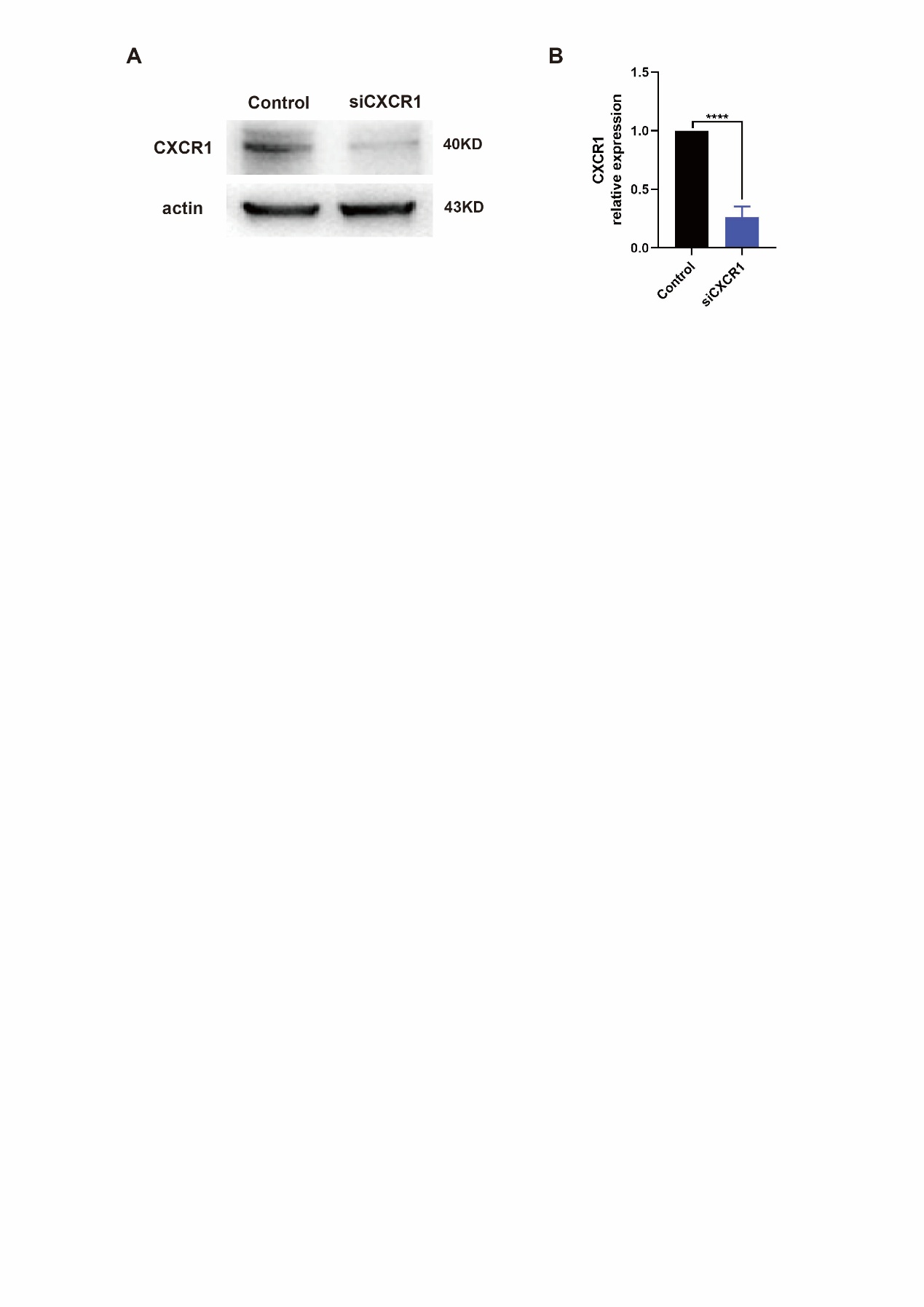


**Supplementary Fig.5** Validation of CXCR1 knockdown in Tregs. (A) Representative western blot image of CXCR1 expression in Tregs. (B) Quantification of CXCR1 expression levels normalized to actin. Control, Tregs cultured alone; siCXCR1, Tregs treated with siCXCR1. N=6. *****p* < 0.0001.


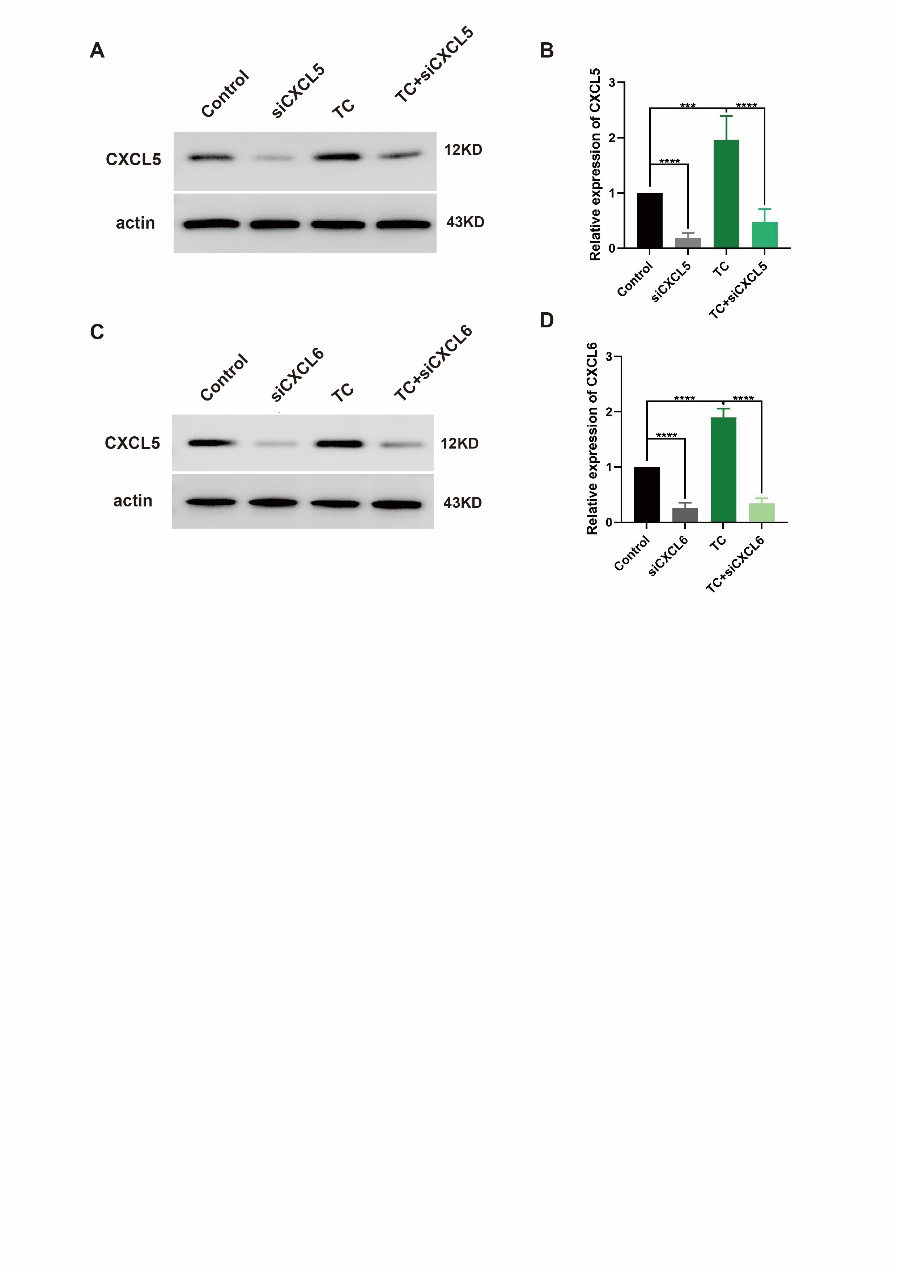


**Supplementary Fig. 6** Validation of CXCL5 and CXCL6 knockdown in MSCs under different treatments. (A) Representative western blot image of CXCL5 expression in MSCs. (B) Quantification of CXCL5 expression levels normalized to actin. (C) Representative western blot image of CXCL6 expression in MSCs. (D) Quantification of CXCL6 expression levels normalized to actin. Control, MSC cultured alone; siCXCL5, MSCs treated with siCXCL5; siCXCL6, MSCs treated with siCXCL6; TC, MSCs pre-conditioned with supernatant from hypoxia-cultured TCs; TC+siCXCL5, MSCs pre-conditioned with supernatant from hypoxia-cultured TCs and treated with siCXCL5; TC+siCXCL6, MSCs pre-conditioned with supernatant from hypoxia-cultured TCs and treated with siCXCL6. N=6. ****p* < 0.001, *****p* < 0.0001.


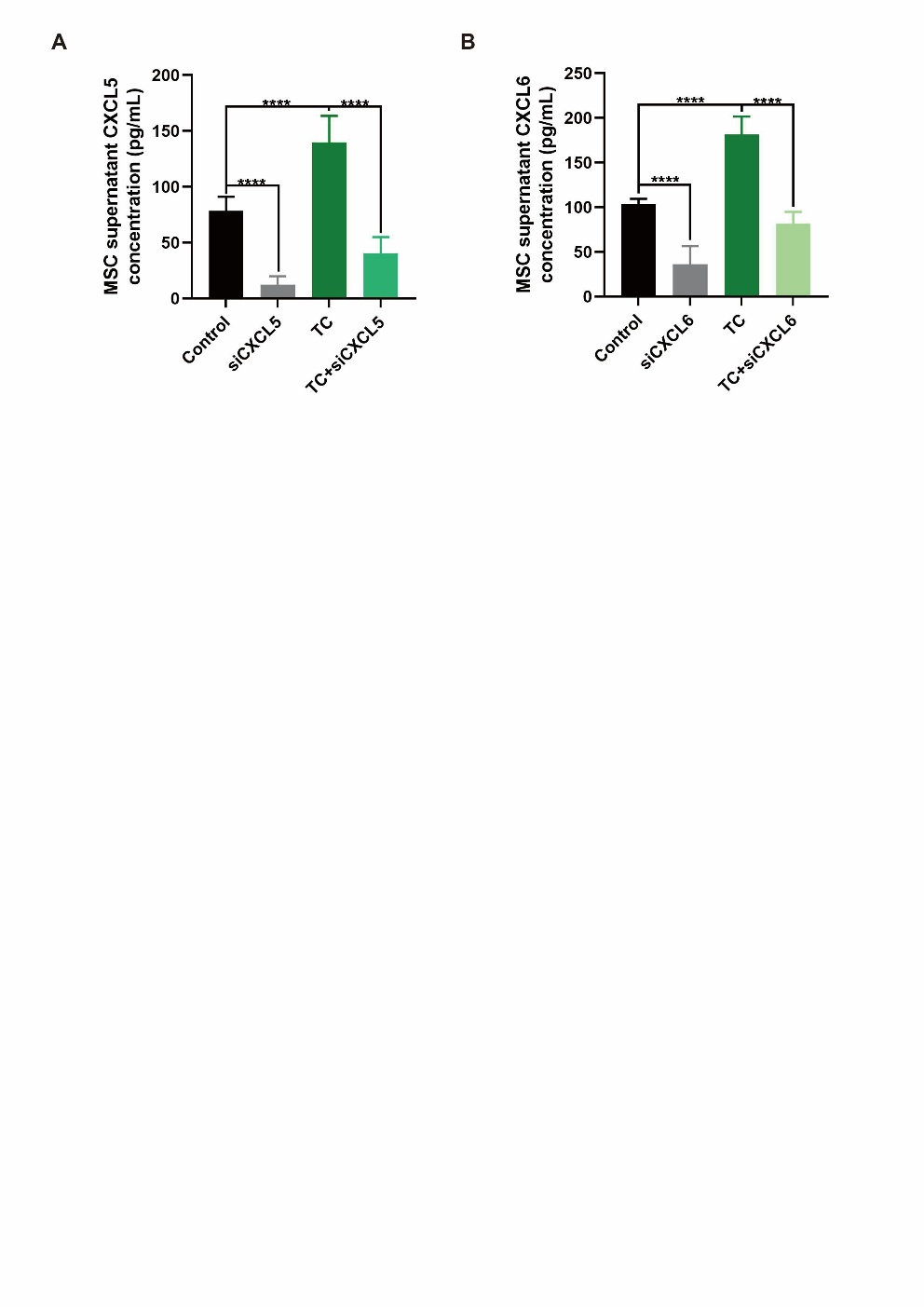


**Supplementary Fig. 7** ELISA detection of CXCL5 and CXCL6 in the conditioned medium from MSCs under different treatments. (A) Concentration of CXCL5 in the conditioned medium from MSC. (B) Concentration of CXCL6 in the conditioned medium from MSC. Control, MSC cultured alone; siCXCL5, MSCs treated with siCXCL5; siCXCL6, MSCs treated with siCXCL6; TC, MSCs pre-conditioned with supernatant from hypoxia-cultured TCs; TC+siCXCL5, MSCs pre-conditioned with supernatant from hypoxia-cultured TCs and treated with siCXCL5; TC+siCXCL6, MSCs pre-conditioned with supernatant from hypoxia-cultured TCs and treated with siCXCL6. N=6. *****p* < 0.0001.
